# Magneto‐Responsive Hybrid Layered Double Hydroxides With Improved Electrochemical Performance and Field‐Actuated Healing

**DOI:** 10.1002/smsc.70335

**Published:** 2026-07-07

**Authors:** Jeena Mariya Sebastian, Karthik Kiran Sarigamala

**Affiliations:** ^1^ CO_2_ Research and Green Technologies Centre Vellore Institute of Technology Vellore Tamil Nadu India; ^2^ School of Advanced Sciences Vellore Institute of Technology Vellore Tamil Nadu India

**Keywords:** cycle life, hybrid supercapacitor, layered double hydroxides, magnetic field, magneto‐healing

## Abstract

Overcoming the intrinsic transport limitations of hybrid supercapacitors using magneto stimulus‐responsive layered double hydroxide (LDH) materials is a promising strategy. Here, we present an urchin‐like Ni–Co LDH@CNT hybrid that exhibits extraordinary field‐enhanced capacitance under a relatively low magnetic field‐strengths. The presence of Ni^2+^/Ni^3+^ and Co^2+^/Co^3+^ allows fast electron hopping and increases the magnetic coupling, while the defects‐rich CNTs promote charge delocalization and fast charge transfer. The magnetohydrodynamic microconvection induced by Lorentz forces enhances the ion permeation and surface‐controlled redox activity at the hybrid interface. The ion diffusion coefficient increases 2.3‐fold (∼6.53 × 10^−13^ cm^2^ s^−1^), while the Tafel slope declined from 77.62 to 62.23 mV dec^−1^ under field, signifying faster reaction kinetics. The Ni–Co LDH@CNT delivers a high‐specific capacity of 657.8 mC cm^−2^ at 1 mA cm^−2^ with 78.2% improvement over zero‐field conditions and retains ∼98% capacity retention even after 3000 cycles. Magnetic measurements reveal that the 60 mT cycled electrode shows high saturation magnetization of 1.74 emu g^−1^ compared to the zero‐field (1.45 emu g^−1^), indicating an increased spin alignment and stronger magnetic coupling that facilitates electron transport and redox activity. An asymmetric hybrid device prototype demonstrates reversible field‐driven capacity recovery over 10 000 cycles, illustrating magnetically induced self‐recovery behavior.

## Graphical abstract

The work describes magnetic‐field regulated charge transport and interfacial kinetics at the LDH‐based hybrid electrode, enabling a low activation barrier for redox reactions and accelerating electron‐transfer processes.  The synergistic magneto‐electrochemical coupling in the hybrid material can significantly promote the charge–discharge dynamics of the supercapacitor and enhance the energy storage performance.

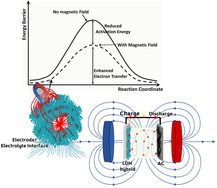

## 1 Introduction

The rising energy demand for electrified transportation and data centers has intensified research on hybrid supercapacitors (HSCs) [1, 2, 3] due to their ability to stabilize and enhance power management. In HSCs, the faradic charge storage mechanism of a battery‐type electrode provides high energy density, and the rapid‐discharge capability is provided from the capacitive‐type electrodes [4, 5, 6, 7]. However, the poor cycle‐life stability of battery‐type materials, limited energy density relative to standalone batteries, and the complex electrode–electrolyte interactions significantly reduce the overall performance of HSCs [8, 9, 10]. Recently, it has been explored that these limitations can be addressed by intricate use of stimuli‐responsive energy materials, which can be sensitive to light or magnetic fields. The use of external stimuli can enhance the battery‐type electrode performance by improving the ion transport, electron transfer rate, and interfacial kinetics [11, 12, 13, 14, 15].

In the context of magnetic field‐assisted strategies, the charge–discharge kinetics in HSCs can be improved when ion transport, pseudocapacitive reactions, and electron transfer are promoted [16, 17, 18, 19, 20, 21]. The hydrodynamic behavior of the electrolyte can be altered by the combined action of electric and magnetic fields, thereby modifying the electrical properties of electrodes, electrolytes, and their interfaces. This phenomenon between magnetic fields and the ionically conductive fluids is explained by magnetohydrodynamic (MHD) effects [17]. The Lorentz force (*F*_L_) plays a crucial role in these systems with enhanced mass transport, ion diffusion, and gas bubble removal, which in turn promotes the overall cell performance. The electromagnetic interactions by Faraday's law of induction, Ampere's law, and the Lorentz forces result in the response of conductive but nonmagnetic electrolytes [18, 19]. The movement of the conducting fluid within the magnetic field induces an electromotive force (Faraday's law), whereas the resulting current generates a secondary magnetic field that couples with the applied field, and the resulting Lorentz force drives enhanced fluid motion, improving the electrochemical performance [20, 21].

These effects in carbon‐based electrodes are confirmed with previous reported experimental investigations. For instance, Zhu et al. showed that applying an external magnetic field enhanced the capacitance of graphene and magnetic graphene nanocomposites without altering their electronic structure [22]. Similarly, Wang et al. and Pal et al. reported enhanced electrochemical performance in activated carbon/Fe_3_O_4_ and Fe_3_O_4_/rGO composites, emphasizing the combined effect of external magnetic fields and intrinsic magnetic properties of battery‐type electrode materials. The improved performance was attributed to faster electron transfer, higher interfacial charge density, efficient ion transport and improved cation intercalation‐deintercalation within the battery‐type material [15, 23]. A fundamental understanding of the magnetic behavior of battery‐type materials is important for HSCs, as their response to external fields is governed by electron spin, orbital motion, and exchange interactions [24]. It is also realized that the ferromagnetic materials with aligned magnetic moments and high permeability retain magnetization even after the field is removed, which helps in ion migration and reducing interfacial resistance in HSCs [25, 26].

Layered double hydroxides (LDHs) are an attractive choice for preparing hybrid materials for supercapacitors, which are composed of positively charged hydroxide layers intercalated with anions and solvent molecules, exhibiting tunable magnetism through control of composition, morphology, and interlayer spacing [27]. However, their performance is constrained by low electrical conductivity and structural degradation while cycling, thereby decreasing the power density and stability [28, 29, 30]. Nevertheless, Ni–Co layered double hydroxide (LDH) is still considered as a fascinating and high‐potential material due to the intrinsic spin moments of Ni^2+^/Ni^3+^ and Co^2+^/Co^3+^ ions. These spin moments arise from unpaired d‐orbital electrons of the in‐plane super exchange and out‐of‐plane dipolar interactions [27]. Under the influence of an external magnetic field, these moments align, inducing spin‐dependent electronic splitting, promoting spin‐selective electron transfer, and lowering the activation energy for charge transport [31]. Altogether, these effects improve ion migration, ion–water interactions and electrode–electrolyte dynamics, thereby enhancing overall charge storage [11]. In LDH‐based hybrid magnetic electrodes, employing carbon nanotubes (CNTs) plays a crucial role in improving their performance. The CNT's magnetic properties arise from the localized unpaired spins of carbon atoms, structural vacancies, line defects, and interaction with magnetic compounds [32, 33, 34, 35, 36]. Ferromagnetism with magnetic moments ranging from 0.3 to 0.8 µB, can arise from carbon atoms near defect sites, such as monovacancies that form pentagonal rings. At the same time, divacancies often reconstruct into octagonal geometries, thereby eliminating the associated magnetic moment [36]. As LDH‐based magnetic materials respond to external magnetic fields, their magnetic properties can be further improved by combining with magnetic CNTs, resulting in the development of functional magnetic hybrids.

In order to study this concept, we designed Ni–Co LDH@CNT hybrid as a model system to investigate the synergistic effects of LDHs with conductive carbon nanostructures for HSC application. The integrations of CNTs improve the electronic conductivity and facilitate efficient ion transport, while the Ni–Co LDH provides high pseudocapacitive activity through reversible faradaic reactions. Electrochemical characterization under the influence of an external magnetic field was employed to probe field‐induced changes in terms of charge transfer, ion diffusion, and redox kinetics, including magneto‐ionic modulation, which describes the magnetic‐field‐driven switching and enhancement of ion mobility within the electrode–electrolyte interface. The Ni–Co LDH@CNT electrode exhibited an exceptional 78.2% increase in specific capacitance, rising from 738 to 1315.6 mF cm^−2^ (specific capacity: 368.9–657.8 mC cm^−2,^ respectively) at a current density of 1 mA cm^−2^ under the influence of an external magnetic field. The electrochemical impedance analysis reveals a ∼2.3‐fold increase in ion diffusion coefficient (2.85 × 10^−13^ to 6.53 × 10^−13^ cm^2^ s^−1^), confirming enhanced ion transport upon application of an external magnetic field. The Tafel analysis demonstrates a synergistic interaction between magnetic‐field‐assisted charge transport and activation of electrochemically active sites, leading to enhanced ion accessibility and accelerated redox kinetics.

Further, the large improvement driven by the external field effect was also supported by extensive investigations conducted during electrode postmortem analysis under no field/applied field conditions. The ex‐situ characterizations were performed on the Ni–Co LDH@CNT cycled electrodes; especially, the superconducting quantum interference device (SQUID) measurements revealed soft ferromagnetic behavior of the hybrid, which is characterized by low coercivity and remanence, indicating near‐reversible magnetization. The electrode cycled under a 60 mT magnetic‐field‐induced operating conditions exhibited a higher saturation magnetization of 1.741 emu g^−1^ compared with 1.452 emu g^−1^ for the electrode cycled without magnetic‐field exposure. Additionally, at low temperatures (5 K), enhanced magnetic domain interactions and improved spin alignment are evident. The MHD effects also significantly improved the specific capacity and long‐term cycling stability of the fabricated asymmetric hybrid device. The long‐term cycling studies reveal a reversible field‐assisted performance recovery mechanism (magneto‐healing) rather than permanent structural regeneration, producing a field actuated‐healing‐like electrochemical response through dynamic optimization of the electrode/electrolyte interface. This work provides a strong foundation for the rational design of multifunctional and magneto‐responsive hybrid electrode materials.

## 2 Results and Discussion

### 2.1 Structure, Composition, and Morphological Analysis

**FIGURE 1:**
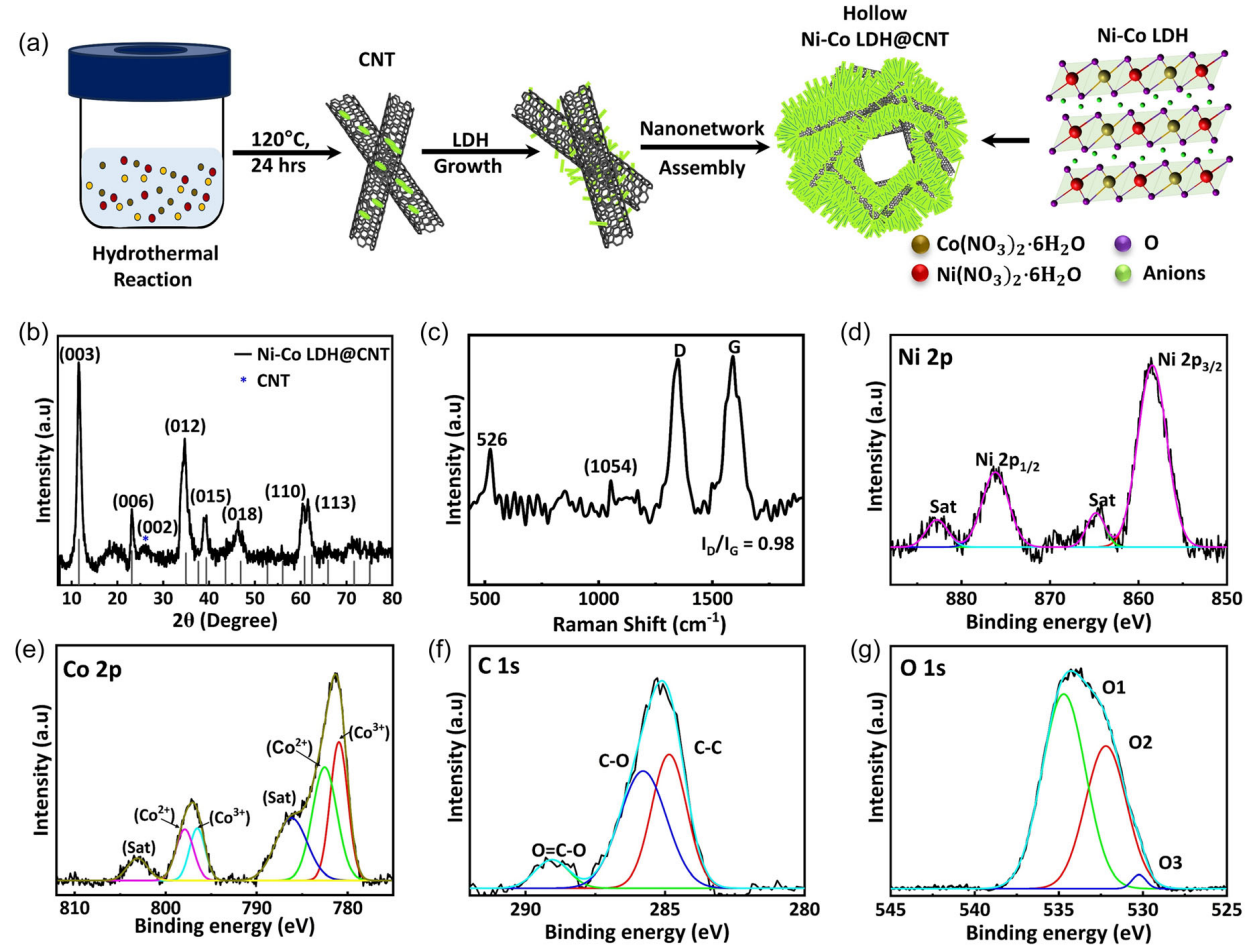
(a) Schematic illustration of the synthesis process of Ni–Co LDH@CNT hybrid. Structural and chemical composition analysis of Ni–Co LDH@CNT. (b) XRD pattern confirming the crystalline phase of Ni–Co LDH@CNT. (c) Raman spectrum showing characteristic carbon bands and the *I*_(D)_/*I*_(G)_ ratio. High‐resolution XPS spectra: (d) Ni 2p, (e) Co 2p, (f) C 1s, and (g) O 1s, respectively.

A hollow urchin‐like intertwined framework of Ni–Co LDH@CNT was obtained by growing Ni–Co LDH nano‐spines onto CNT fibers. The unique open porous features were resultant from the combined influence of the Kirkendall effect and Ostwald ripening process [37, 38]. Initially, the Ni^2+^ and Co^2+^ ions undergo hydrolysis and nucleation on the CNT surface, forming a uniform Ni–Co hydroxide layer. As the reaction proceeds, the unequal diffusion rates of Ni and Co ions result in the generation of vacancies at the core–shell interface, which later coalesce to create internal voids, a phenomenon explained by the Kirkendall effect. Simultaneously, Ostwald ripening occurs, where less stable nanoparticles dissolve and redeposit on the surface of crystallites, resulting in a more thermodynamically stable system [37, 38, 39, 40, 41]. The combined action of these two processes results in the formation of a well‐defined open porous Ni–Co LDH framework on CNT, as shown in Figure 1a.

The proposed architecture was initially studied using X‐ray diffraction (XRD) analysis. The Ni–Co LDH@CNT exhibited a rhombohedral crystal structure, which was confirmed by the distinct sharp peaks that indicate high crystallinity (Figure 1b), with the Bragg's reflections corresponding to (003), (006), (012), (015), (018), (110), and (113) planes. These reflections suggest the formation of a typical hydrotalcite‐like structure with an interlayer spacing of 0.904 nm (corresponding to d_(003)_). The CNT incorporation into the backbone of the LDH nanostructure was further confirmed by the (002) plane. Raman spectroscopy is used to corroborate the XRD data for confirming the successful integration of conductive CNT with the active material. Figure 1c shows the Raman spectrum of the synthesized material, showing an *I*_(D)_/*I*_(G)_ ratio of 0.98, indicating a moderate level of structural disorder induced within the carbon framework. The formation of a hybrid composite often results in disorder due to the vacancies associated with the edge sites or functional groups. These defects enhance the electrochemical performance by providing additional active sites for charge storage while still maintaining sufficient graphitic domains for electron transport. The sharp peaks at 526 and 1054 cm^−1^ are attributed to the vibrations of metal oxide bonds (M–O, O–M–O, and M–O–M, where M represents Ni and Co), as well as the C–O vibration mode, respectively [42]. The Fourier transform infrared spectroscopy (FTIR) analysis (Figure S1) of Ni–Co LDH and Ni–Co LDH@CNT show characteristic bonds corresponding to O–H stretching, C–O vibrations, and metal–oxygen/hydroxide (Ni—OH, Co—O) bonds, confirming the presence of typical functional groups in the LDH structure.

The composition and valence states of elements were analyzed using X‐ray photoelectron spectroscopy (XPS) for both Ni–Co LDH (Figure S2) and Ni–Co LDH@CNT samples (Figure 1d–g). The XPS spectra confirm the presence of Ni, Co, C, and O in Ni–Co LDH@CNT hybrid with no additional foreign elements. The successful incorporation of carbon and oxygenated functionalities was confirmed by the distinct C 1s and O 1s signals, along with characteristic Ni 2p and Co 2p peaks of LDH. The deconvoluted Ni 2p spectrum in Figure 1d shows two distinct peaks at 858.3 and 876.16 eV, corresponding to Ni 2p_3/2_ and Ni 2p_1/2_, respectively. Additionally, two shake‐up satellite peaks (sat) were observed at 865.7 and 882.7 eV, which are the characteristic peaks of Ni^2+^ ions. Figure 1e shows the deconvoluted XPS spectrum of Co 2p, which comprises two spin–orbit doublets and two sat peaks corresponding to the Co^3+^ and Co^2+^ oxidation states. The two prominent peaks at 780.9 and 796.5 eV are assigned to the Co^3+^ and a pair of peaks at 782.5 and 797.9 eV corresponds to Co^2+^ species, and two satellite peaks at 786 and 803 eV, respectively. This confirms the presence of two oxidation states (Co^3+^ and Co^2+^) in the hybrid. The C 1s spectrum (Figure 1f) can be deconvoluted into three peaks located at 284.8, 285.7 and 289 eV, which correspond to C–C, C–O, and O=C–O functional groups, respectively. Similarly, the O 1s spectrum (Figure 1g) can be deconvoluted into three distinct peaks at 530.7, 532.2, and 534.7 eV, denoted as O3, O2, and O1, respectively. This shows the coexistence of surface oxygenated groups and absorbed species, which contribute to improved interfacial interactions [43, 44].

**FIGURE 2:**
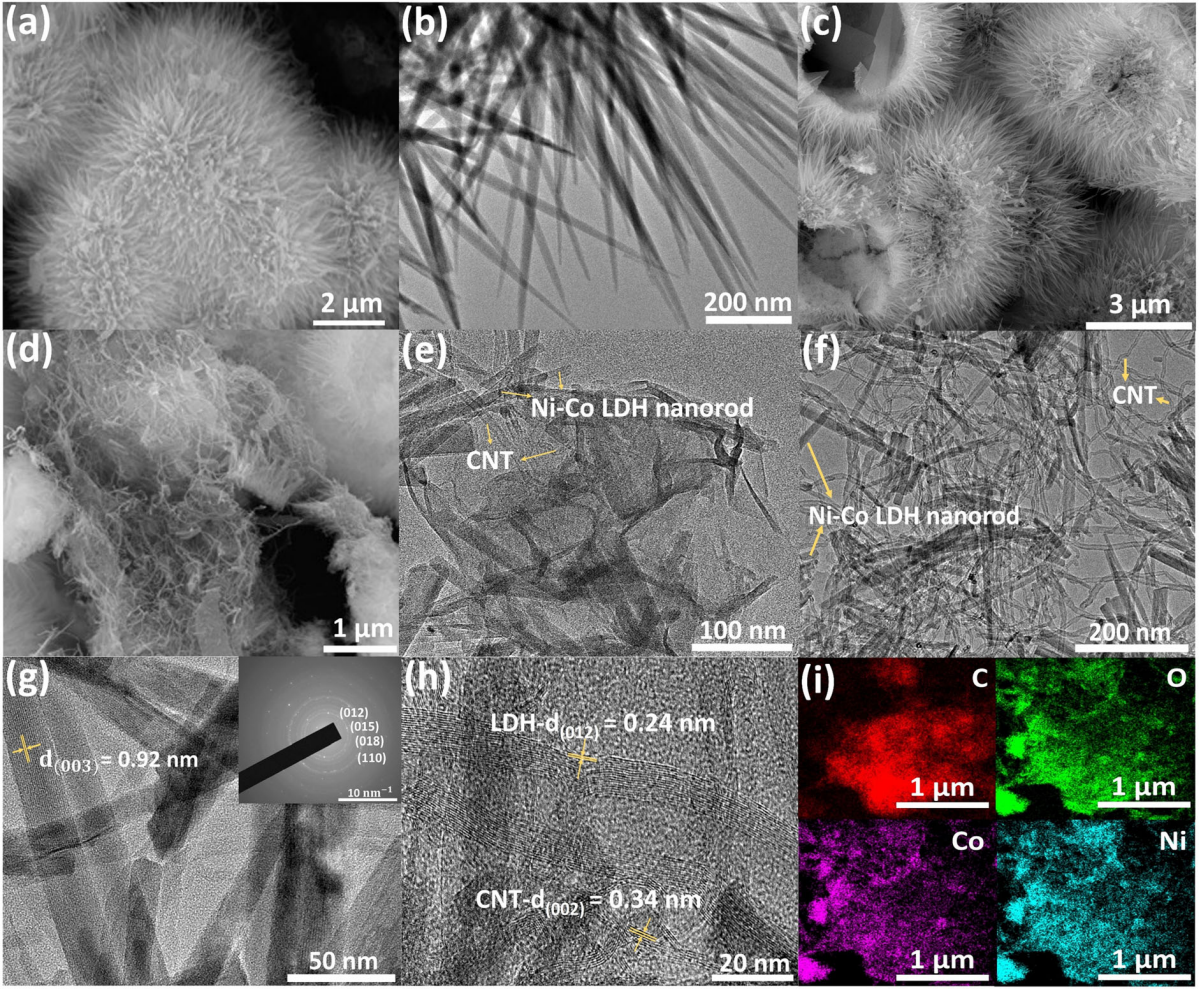
FE‐SEM micrograph revealing the surface morphologies: (a) Pristine Ni–Co LDH, (c) and (d) Ni–Co LDH@CNT. TEM images: (b) Pristine Ni–Co LDH, (e) and (f) Ni–Co LDH@CNT. (g) Lattice fringes of LDH lattice in the hybrid material. Inset‐ SAED patterns. (h) Lattice fringes of hybrid showing both Ni–Co LDH and CNT in an intertwined architecture. (i) EDAX‐elemental mappings of C, O, Co, and Ni on STEM image, respectively.

Field emission‐scanning electron microscopy (FE‐SEM) was used to characterize the surface morphologies of the synthesized pristine and hybrid samples. Figure 2a shows a distinctive spherical nanostructure of pristine Ni–Co LDH, characterized by a dense arrangement of nano needle‐like structures. These nanoneedles have a maximum diameter of 40 (±5) nm with a resemblance to the sea urchin spines, which are self‐assembled into aggregated nanoclusters. Figure 2c,d show the FE‐SEM micrographs of Ni–Co LDH@CNT, with external surface features displaying urchin‐like spine structures assembled from Ni–Co LDH, being successfully nucleated and grown directly on the surface of the CNT skeleton. The micrographs display an intertwined hybrid network architecture with hollow interior morphology, which is successfully formed with CNTs serving as a conductive backbone and the LDH nanoneedles as active metallic sites. The intertwined nanostructure offers a high surface area and efficient ion diffusion pathways beneficial for electrochemical performance. For a better understanding of the internal microstructure of the hollow urchin‐like spiny framework of Ni–Co LDH@CNT, transmission electron microscopy (TEM) analysis was performed. Figure 2b shows a cluster of sharp nano‐needle‐like structures of Ni–Co LDH. The TEM analysis of Ni–Co LDH@CNT from Figure 2e,f also clearly confirm the presence of numerous needle‐like structures of LDH grown on the CNT fibers, demonstrating effective integration of the two materials in a hollow spherical structure. A clear differentiation of CNTs and Ni–Co LDH with an interwoven matrix is marked on the TEM micrographs. The presence of conductive CNT enhances the overall electrical conductivity of the LDH material. Figure 2g shows the lattice fringes with an interplanar spacing of 0.92 nm corresponding to the (003) crystal plane, showing its large interlayer distance. This expanded crystal lattice is crucial for improving the electrochemical performance as it offers wider pathways for rapid and efficient intercalation of charged species, directly leading to improved charge storage capacity and faster kinetics of the supercapacitor. The evaluated d‐spacing from high‐resolution TEM images matched well with the interplanar spacing of Ni–Co LDH@CNT obtained from XRD. The bright spots observed in the selected area electron diffraction (SAED) pattern (Figure 2g‐inset) are also in good agreement with the diffraction pattern. Figure 2h shows a clear distinction of CNT and LDH lattice fringes with an interplanar spacing of 0.24 nm corresponding to the (012) plane of Ni–Co LDH, and 0.34 nm for the (002) plane of CNT. The efficient structural integration of Ni–Co LDH and CNTs can be observed by the close interfacial contact, which helps to boost the electron transport for synergistic energy storage performance. The energy‐dispersive X‐ray spectroscopy (EDAX) elemental mappings of Ni–Co LDH@CNT obtained from TEM (Figure 2i) show a uniform distribution of C, O, Co, and Ni mapped over the STEM image, confirming nanonetworks of Ni–Co LDH structure homogeneously anchored on the CNT backbone.

### 2.2 Energy Storage Performance and the Effect of the Magnetic Field

**FIGURE 3:**
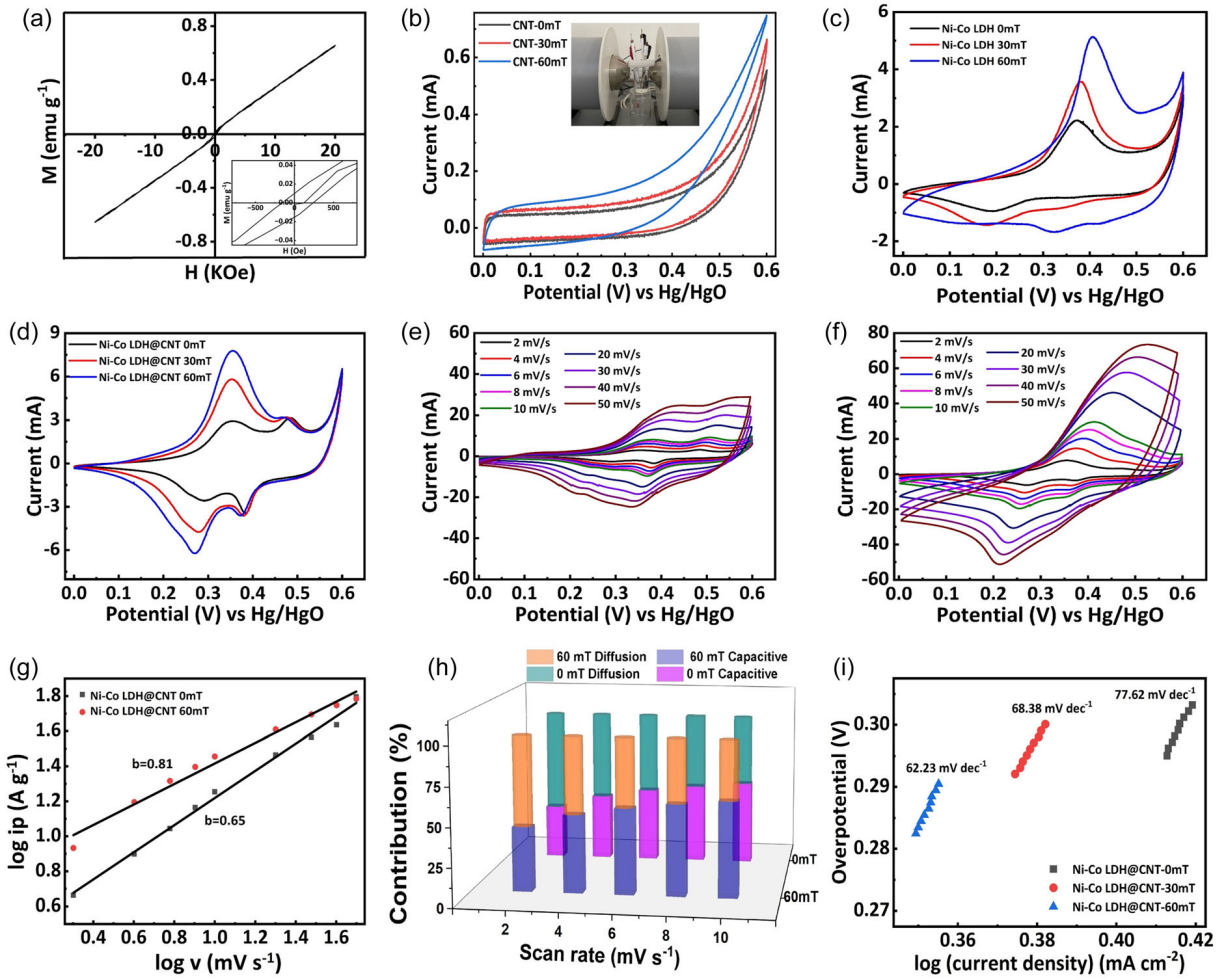
(a) Magnetization versus magnetic field (M‐H) curve of Ni–Co LDH@CNT at room temperature (the inset shows a magnified view of the M‐H graph from −800 to +800 Oe). The effect of the magnetic field strength on the CV profiles in 3 M KOH: (b) CNT (the inset shows the magnetic field‐assisted electrochemical setup), (c) Ni–Co LDH, (d) Ni–Co LDH@CNT, (e) and (f) CV curves obtained at various scan rates for Ni–Co LDH@CNT at applied fields of 0 and 60 mT, respectively. Comparison of electrochemical data for Ni–Co LDH@CNT at no field and applied field conditions: (g) Log (Scan rate) versus log (peak current) plot. (h) Bar chart showing diffusion and capacitive contributions at various scan rates. (i) Tafel plot.

The electrochemical behavior of pristine and hybrid LDH in a magnetic field was systematically analyzed by their comparative performance parameters evaluated at zero‐field and applied‐field conditions. These studies were conducted in an in‐house developed magneto‐electrochemical set‐up (as displayed in the inset of Figure 3b) with adjustable external magnetic field strengths (0–100 mT) to understand the magnetization effects on electrodes, and the results are presented in the following sections. The specific field intensities used here were optimized‐based on our experimental setup requirements.

Initially, using a vibrating sample magnetometer, the magnetic properties of the CNT, pristine LDH (Figure S4) and hybrid LDH (Figure 3a) samples were measured at ambient temperature by varying the field in the range from −20 to +20 kOe, and the results were compiled in Table 1. The CNTs used for hybrid material preparation exhibits a coercivity (*H*_c_) of 153 Oe, a remanence (*M*_r_) of 0.14 emu g^−1^, and a saturation magnetization (*M*_s_) of 1.4 emu g^−1^, while the prepared pristine Ni–Co LDH demonstrates an ultra‐soft ferromagnetism with a *H*_c_ of 101 Oe, and *M*_s_ of 1.68 emu g^−1^. On the contrary, the Ni–Co LDH@CNT hybrid has also exhibited similar soft ferromagnetic properties, with the highest *H*_c_, but lower *M*_s_ as revealed by a narrow hysteresis loop (Figure 3a). These magnetic parameters indicate that incorporating CNTs into Ni–Co LDH modifies the magnetic response of the material. The Ni–Co LDH@CNT hybrid exhibits the highest coercivity, implying stronger resistance to magnetic reversal, likely due to interfacial coupling and restricted motion of magnetic domains at the LDH‐CNT interface. These differences correspond to variations in particle size, shape, and crystal orientation. The reduced saturation magnetization is likely attributable to CNTs and structural effects [45].

**TABLE 1:** Magnetic property comparison of CNT, Ni–Co LDH, and Ni–Co LDH@CNT materials.

Magnetic properties	CNT	Ni–Co LDH	Ni–Co LDH@CNT
Coercivity (Oe)	153	101	185
Remanence (emu g^−1^)	0.14	0.045	0.094
Magnetic saturation (emu g^−1^)	1.4	1.68	1.25

Importantly, the presence of defects in Ni–Co LDH@CNT maintains the ferromagnetic character, in addition to structural scaffold morphology and abundant active sites in the system. The CNTs also enable effective dispersion and reduced aggregation of the LDH layers. Such interaction probably promotes magnetic coupling of nickel and cobalt ions because of interfacial effects leading to magnetic stability and uniformity [32, 33, 34, 35, 36]. These magnetic properties are likely to influence the electrochemical behavior when exposed to a magnetic field.

Prior to analyzing the electrochemical performance of Ni–Co LDH@CNT hybrid material, we have investigated the individual components, CNT and pristine LDH, using both zero‐field and applied‐field measurements to gain insight into their individual electrochemical contributions. The cyclic voltammetry (CV) analysis of the CNT electrode in the absence and presence of a magnetic fields (0, 30, and 60 mT) for the scan rates of 2–50 mV s^−1^ (Figure S5) was initially performed. As shown in Figure 3b, the CV curves reveal a gradual increase in the enclosed area with increasing magnetic field strength, which signifies an improvement in charge storage. The Galvanostatic charge–discharge (GCD) analysis (Figures S6 and S7a) also strengthens the trend observed in the CV analysis and further clarifies the underlying mechanism. The subtle changes in discharge time and nearly similar capacitance (∼20 mF cm^−2^ at 0.25 to 1 mA cm^−2^) support the conclusion that the intrinsic charge storage functionality of the pristine CNT electrode is unaltered by the magnetic field. The fact that charging time is greatly reduced (22.3% at 60 mT) suggests that the system attains a charge state faster under the applied field. This is consistent with the CV that showed an increase in area, confirming enhanced charge dynamics instead of a change in storage capacity. This interpretation is also reinforced by electrochemical impedance spectroscopy (EIS) findings (Figure S7b). The magnetic field application exhibited an equivalent lowered solution resistance (*R*_s_: 1.109 → 1.059 Ω) and charge transfer resistance (*R*_ct_: 58.49 → 46.32 Ω), indicating increased ionic conductivity and significantly rapid interfacial charge transfer.

The Figure 3c,d display comparative CV profiles of the pristine and hybrid materials at various magnetic field strengths over the potential range of 0–0.6 V. It is interesting to note that both pristine and hybrid materials exhibited strong redox peaks in their CV curves and an improvement in the area enclosed by the CV curve under the external magnetic field. However, under varying magnetic field strengths, the CV profiles of Ni–Co LDH (Figure 3c) display the anodic peak shifts: from 0.372 to 0.407 V and the cathodic peak shifts from 0.188 to 0.319 V as the field increases from 0 to 60 mT. The resulting increase in peak separation reflects quasi‐reversible behavior and relatively slow charge transfer. In contrast, the Ni–Co LDH@CNT electrode (Figure 3d) demonstrates a more pronounced enhancement in electrochemical response with increasing magnetic field strength. Specifically, the anodic peak current increases from ∼3 mA at 0 mT to ∼8 mA at 60 mT, corresponding to an approximately 2.6‐fold enhancement, while the cathodic current exhibits a 2.4‐fold increase. Notably, the absence of any significant shift in peak potentials suggests improved reversibility and accelerated redox kinetics in the hybrid system. Furthermore, scan rate‐dependent CV measurements (2–50 mV s^−1^) of Ni–Co LDH@CNT were also conducted in different field conditions such as 0 mT (Figure 3e) and 30 mT (Figure S8a) revealing a systematic increase in peak current with increasing scan rate, accompanied by a gradual widening of peak separation, indicative of pseudocapacitive behavior with emerging kinetic limitations at higher scan rates due limited ion diffusion and interfacial charge–transfer kinetic at faster potential sweeps. Upon application of a 60 mT magnetic field (Figure 3f), the peak currents across all scan rates are consistently higher, and the enclosed CV area is significantly increased suggesting improved pseudocapacitor behavior compared to the rest. In comparison, the pristine Ni–Co LDH (Figure S9) exhibits only marginal improvement under similar magnetic field conditions, suggesting a comparatively limited influence on its intrinsic electrochemical kinetics. Overall, the enhanced electrochemical performance of Ni–Co LDH@CNT is quantitatively evidenced by the substantial increase in peak currents and CV area, reflecting improved charge transfer and ion transport characteristics. Therefore, it is evident that, when the LDH is integrated with CNTs, the resultant Ni–Co LDH hybrid not only maintains its intrinsic ferromagnetic properties but also takes advantage of the conductive network and structural support provided by CNTs.

To elucidate the enhanced performance observed in CV measurements, a detailed kinetic analysis of the charge storage mechanism was performed for both pristine and hybrid Ni–Co LDH samples. Figures 3g and S10 show the log–log plots of peak current (log *i*_p_) versus scan rate (log *v*). The relationship between the peak current (*i*_p_) and scan rate (*v*) was analyzed using the power‐law equation,

(1) $$i_{p}=\;av^{b}$$

(2) $$\log\;(i_{p})=b\log\;(v)+\log\;(a)$$

Here, the *b*‐value shows the difference between different charge storage processes [46, 47]. The *b*‐value was obtained from the slope of the log (*i*_p_) versus log (*v*) plot. Typically, a *b*‐value of 0.5 indicates a diffusion‐controlled process, while a value approaching 1.0 corresponds to a surface‐controlled (capacitive) mechanism. Here, the *b*‐value was found to increase from 0.65 at 0 mT to 0.81 at 60 mT. This significant shift of the *b*‐value indicates that the magnetic field promotes a more surface‐controlled charge storage mechanism over a diffusion‐controlled one. With variation in scan rate, the charge storage behavior is also affected and can generally be categorized into two types namely, surface‐controlled and diffusion‐controlled processes. To further quantify this, the total current at a specific potential *i* (*V*) was separated into its capacitive (*k*_1_ *v*) and diffusion‐controlled (*k*_2_ *v* ^1/2^) contributions using the Dunns method relation [48, 49, 50].

(3) $$i(V)\;=\;k_{1}v\;+\;k_{2}v^{1/2}$$

For each potential, *k*_1_ and *k*_2_ were determined by rearranging Equation (3) and performing linear fitting. This approach allows quantitative estimation of the relative contributions at different scan rates. The capacitive vs. diffusion‐controlled contributions for Ni–Co LDH@CNT at 0 and 60 mT at various scan rates (from 2 to 10 mVs^−1^) are provided in Figure 3h. At 0 mT, the capacitive contribution increases significantly from 34.74% to 54.34% with increasing scan rate, while the diffusion‐controlled contribution correspondingly decreases from 65.25% to 45.65%. A similar trend is observed under the applied magnetic field of 60 mT, where the capacitive contribution rises from 41.7% to 61.5%, accompanied by a decrease in diffusion‐controlled contribution from 58.2% to 38.46%. This behavior indicates that, at higher scan rates, the charge storage process becomes increasingly dominated by surface‐controlled kinetics in both cases. This increased capacitive contribution supports the previous observation of an increased *b*‐value (closer to 1) under the magnetic field condition.

Further, the Tafel slopes obtained from the linear polarization curves were extracted by fitting the linear region in the high‐overpotential regime, where the reaction is predominantly governed by charge–transfer kinetics. These slopes quantitatively represent the increase in overpotential required for a ten‐fold (one decade) increase in current density and thus serve as important indicators of the electrocatalytic efficiency of the electrodes. The Tafel analysis for CNT, Ni–Co LDH, and Ni–Co LDH@CNT electrodes is presented in Figure S11a, b, and Figure 3i, respectively. For the Ni–Co LDH@CNT electrode, the Tafel slope decreases from 77.62 mV dec^−1^ (0 mT) to 62.23 mV dec^−1^ at 60 mT, corresponding to an overall reduction of ∼9%. In comparison, the Ni–Co LDH electrode shows a moderate improvement with a ∼7.76% reduction in slope at 60 mT, while the pristine CNT exhibits a more pronounced decrease of ∼20.78%, although still remaining kinetically inferior to the hybrid system. The detailed values are summarized in Table 2. This suggests that the improved electrocatalytic activity of the LDH materials is due to the enhanced intrinsic activity of the catalytically active sites [51]. Especially, the applied external magnetic field interacts with the spin state of electrons and influences their energy levels to alter the adsorption–desorption kinetics [52]. The Ni–Co LDH@CNT has the best activity among all three materials, and this can be associated with its morphology and intrinsic active sites and oxygen vacancies or defects emerged while CNT incorporation, as it can improve the active area and electronic structure of the catalyst.

**TABLE 2:** Tafel slope comparison of CNT, Ni–Co LDH and Ni–Co LDH@CNT in 0 and 60 mT field conditions.

Material	Slope at 0 mT, mV dec^−1^	Slope at 30 mT, mV dec^−1^	Slope at 60 mT, mV dec^−1^
CNT	163.79	128.96	102.16
Ni–Co LDH	83.42	73.49	63.79
Ni–Co LDH@CNT	77.62	68.38	62.23

**FIGURE 4:**
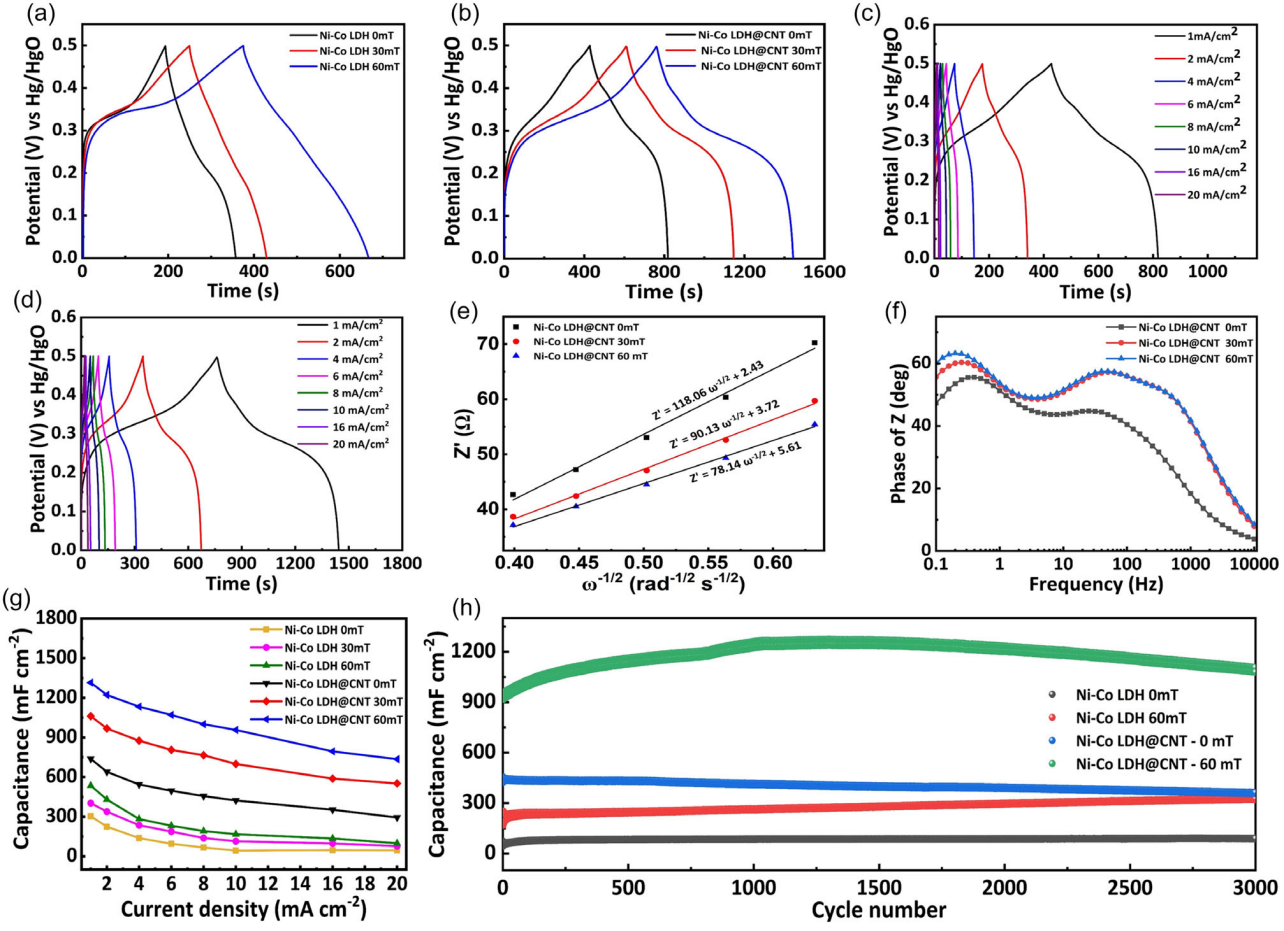
Comparison of GCD profiles: (a) Ni–Co LDH, and (b) Ni–Co LDH@CNT. GCD profiles of Ni–Co LDH@CNT obtained at various current densities under field conditions of (c) 0 mT, and (d) 60 mT. (e) *Z*′ vs *ω* ^−1/2^. (f) Bode plot showing phase angle versus angular frequency for Ni–Co LDH@CNT. (g) Comparison of Specific capacitance at various current densities under three different field conditions. (h) Cycle life comparison plot.

To understand the supercapacitor performance under different external magnetic field strengths, the GCD studies were performed. The Figure 4a shows the GCD comparison plot of Ni–Co LDH at various magnetic field strengths. The discharge specific capacitance increased from 303 to 535 mF cm^−2^ (specific capacity: 151.5 to 267.5 mC cm^−2^) for 0 to 60 mT at a current density of 1 mA cm^−2^. This shows a 76.5% enhancement in capacitance due to the improved ion diffusion to the LDH surface, yielding more surface faradaic reactions and electron transport due to the MHD effect. The pristine material has an asymmetric charge–discharge curve, which supports the earlier discussion on the CV analysis showing its quasi‐reversible nature. In the case of Ni–Co LDH@CNT hybrid (Figure 4b), the specific capacitance value increased from 738 to 1315.6 mF cm^−2^ (specific capacity: 368.9 to 657.8 mC cm^−2^, respectively) under magnetic field strengths ranging from 0 to 60 mT at a current density of 1 mA cm^−2^. This is a remarkable improvement with 78.2% enhancement in specific capacitance at a magnetic field strength of 60 mT. Predominantly, the hybrid material has a more symmetric curve, showing good reversibility and faster ion transport owing to the low internal resistance. The Ni–Co LDH@CNT electrode primarily exhibits faradaic redox reactions due to the insertion and extraction of OH^−^ ions at the electrochemically active sites within the layered structure, where the surface faradic contribution is due to the reversible redox transitions of Ni and Co species [53, 54]. The electrochemical process is governed by the following redox reactions [55]:

(I) $$\mathrm{Ni}{\left(\mathrm{OH}\right)}_{2}+{\mathrm{OH}}^{-}\;⇌\text{NiOOH}+H_{2}O+e^{-}$$

(II)

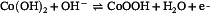

(III) $$\text{CoOOH}+{\mathrm{OH}}^{-}⇌{\mathrm{CoO}}_{2}+H_{2}O+e^{-}$$

In an alkaline electrolyte, Ni(OH)_2_ (Ni^2+^) is oxidized into NiOOH (Ni^3+^), with the release of an electron as in Equation (I). Similarly, Co(OH)_2_ (Co^2+^) initially forms CoOOH (Co^3+^) as shown in Equation (II), and then finally converts to CoO_2_ via Equation (III). These are multielectron redox reactions, in which each step stores/releases charge via electron transfer, contributing to their faradaic nature. Nickel provides high specific capacitance via its Ni(OH)_2_/NiOOH redox couple, while cobalt improves conductivity and rate capability through sequential Co(OH)_2_/CoOOH/CoO_2_ transitions, reducing charge transfer resistance. The integration of CNTs into Ni–Co LDH enhanced electronic conductivity and facilitates efficient ion transport through improved electrolyte accessibility and porous pathways. Additionally, the CNT's high surface area contributes to electric double‐layer capacitance at the electrode–electrolyte interface, while the Ni–Co LDH provides high pseudocapacitive activity through reversible faradaic reactions. This combined effect is clearly understood from the high discharge specific capacitance of Ni–Co LDH@CNT.

Figure 4c shows the GCD behavior of Ni–Co LDH@CNT without the magnetic field at various current densities. The GCD curves exhibit a nonlinear profile with distinct plateaus, indicating battery‐type faradaic charge storage, in which the discharge time and the IR (resistive) drop become more noticeable with increasing current density, reflecting the typical kinetic limitation. Figure 4d shows the GCD behavior of Ni–Co LDH@CNT under 60 mT of external magnetic field, indicating significantly longer discharge duration at all corresponding current densities, showing a much higher specific capacitance. Similarly, GCD curves were also obtained for Ni–Co LDH@CNT at 30 mT and Ni–Co LDH at 0, 30, and 60 mT as shown in Figures S8b and S12, respectively.

To analyze the effect of the applied field on the diffusion of ions in the Ni–Co LDH@CNT electrode–electrolyte interface, a Warburg plot is obtained from the EIS (Figure S14c) in the frequency range of 0.1–10 kHz. The Warburg slopes were evaluated from *Z*′ vs. *ω* ^−1/2^ graph (where *ω* represents lower frequencies) to assess the diffusion coefficients (*D*) as shown in Figure 4e. As can be seen from the plot, the Warburg coefficient (*σ*) decreases significantly from 118.06 to 78.14 Ω s^−1/2^, when exposed to the field, indicating significantly enhanced ion diffusion kinetics. Since the diffusion coefficient is inversely proportional to the square of *σ*, this corresponds to an approximate 2.3‐fold increase in ion diffusivity. Under no external magnetic field, a diffusion coefficient of 2.85 × 10^−13^ cm^2^ s^−1^ is obtained, whereas when exposed to an external magnetic field strength of 60 mT, a remarkable increase is seen as the value reaches 6.53 × 10^−13^ cm^2^ s^−1^. Corroborating this, bode plots (Figure 4f) also reveal a significant phase angle increase at 60 mT. The phase angle is increased from 47.3° to 60.1° for 0–60 mT, respectively. An increase in phase angle toward 90° indicates more capacitive behavior, reflecting that charge storage is predominantly governed by pseudocapacitive reactions. These results align well with the observed increase in *b*‐value and the change in contribution ratios, which show faster charge storage kinetics and better electrochemical access.

A detailed comparison of specific capacitance versus current density for Ni–Co LDH and Ni–Co LDH@CNT across various magnetic field strengths is presented in Figure 4g. For all the cases, the specific capacitance decreases as current density increases, which is usually observed in supercapacitors owing to the incomplete utilization of the active material. Under no magnetic field condition (0 mT), the Ni–Co LDH exhibited a capacitance of 303 mF cm^−2^ (151.5 mC cm^−2^), and Ni–Co LDH@CNT displayed a superior capacitance value of 737.84 mF cm^−2^ (368.92 mC cm^−2^) at 1 mA cm^−2^. On the application of a magnetic field (60 mT), the specific capacitance increased to 535.44 mF cm^−2^ (267.7 mC cm^−2^) for Ni–Co LDH and 1315.6 mF cm^−2^ (657.8 mC cm^−2^) for Ni–Co LDH@CNT, respectively. Overall, the application of an external magnetic field on the interfacial electrode leads to a higher Lorentz force on migrating ions, and the MHD effect intensify electrolyte convection, resulting in enhanced convective charge transfer [17], decrease the diffusion layer thickness at the Ni–Co LDH@CNT electrode–electrolyte interface, and accelerate OH^−^ ion transport [11]. Moreover, since the magnetic moments of Co and Ni 3d orbitals induce spin‐polarized electron transfer, which favors orbital overlap during the redox transitions (Co^2+^/Co^3+^ or Ni^2+^/Ni^3+^) might have also significantly enhanced the pseudocapacitance response of this battery‐type electrode.

Figure 4h illustrates the cycling performance of both Ni–Co LDH and Ni–Co LDH@CNT under 0 and 60 mT field conditions at a current density of 10 mA cm^−2^. At 0 and 60 mT, the Ni–Co LDH@CNT exhibited a significantly higher capacitance of 423 and 956 mF cm^−2^ (211.5 and 478 mC cm^−2^), respectively, compared to the pristine material, which displayed only 44 mF cm^−2^ (22 mC cm^−2^) at 0 mT and 167 mF cm^−2^ (83.5 mC cm^−2^) at 60 mT, respectively. The cycle life profile is almost linear over 3000 cycles, achieving ∼98% specific capacitance retention in all cases. Additionally, under the magnetic field induced condition, the hybrid material shows an enhanced capacitance of 956 mF cm^−2^ (478 mC cm^−2^) and a gradual increase in the specific capacitance value reaching up to 1280 mF cm^−2^ (640 mC cm^−2^) for about ∼1300 cycles before stabilizing at 1080 mF cm^−2^ (540 mC cm^−2^) over 3000 cycles. This shows that the hybrid Ni–Co LDH@CNT still outperforms the field‐free system and the field‐enhanced pristine Ni–Co LDH throughout the cycle life test.

The selection of 60 mT as the optimal threshold magnetic field strength is rigorously substantiated by the electrochemical responses, as shown in Figure S14. The CV profiles in Figure S14a, clearly demonstrate that the electrode operated at 60 mT exhibits the largest integrated area and the highest current density, indicating superior charge‐storage capability and faster faradaic reactions relative to the other investigated field strengths. The GCD curves in Figure S14b further corroborate this trend, as discharge measurements show that the 60 mT condition exhibits the longest discharge duration, indicative of the highest attainable specific capacitance. Moreover, EIS data in Figure S14c reveals that applying 60 mT reduces charge–transfer resistance and enhances ion diffusion, as evidenced by the lower real impedance and steeper Warburg slope. Any further increase in magnetic field strength beyond this threshold (>60 mT) results in a noticeable decline in electrochemical performance as observed with 100 mT field condition. This is expected due to the generation of localized heating, disorder caused in the double layer or even due to the bubble formation in aqueous electrolyte [56]. The applied field strength was also benchmarked against established literature standards to ensure comparability. Table S1 provides a comprehensive comparison of our measured magnetic properties with those reported in prior studies employing varying field strengths (ranging from 1.34 to 100 mT). Therefore, 60 mT represents an optimal regime wherein magnetic‐field‐induced improvements in electron transport, redox kinetics, and ion diffusion are maximized while avoiding the deteriorating effects associated with excessive magnetic field strengths, ultimately resulting in the most favorable overall electrochemical performance.

### 2.3 Mechanism and Ex‐Situ Analysis

**FIGURE 5:**
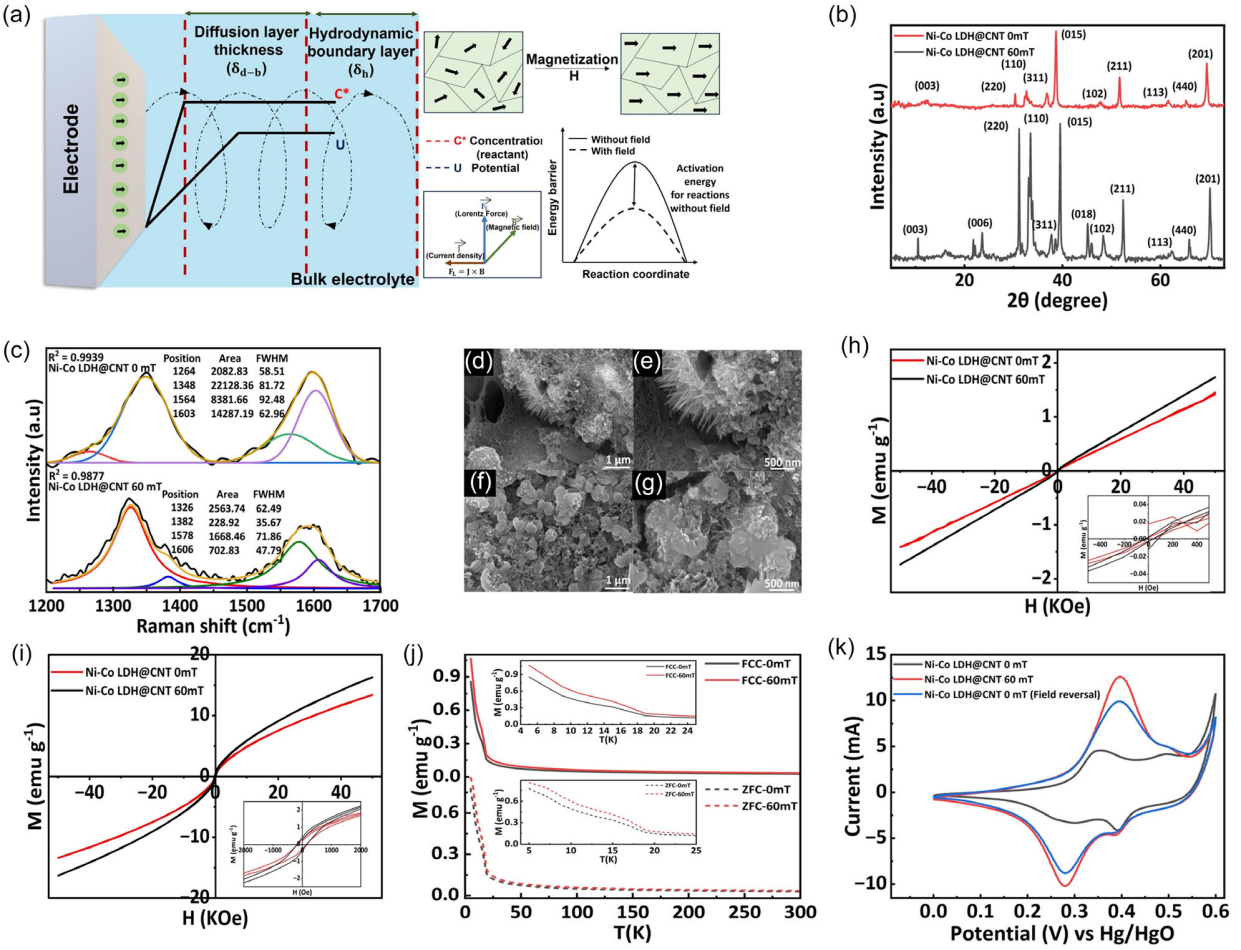
(a) A schematic illustration of the magneto‐electrochemical effect on the Ni–Co LDH@CNT electrode. Ex situ analysis of Ni–Co LDH@CNT: (b) XRD patterns. (c) Deconvoluted Raman spectra. FE‐SEM images showing post‐mortem analysis of cycled Ni–Co LDH@CNT electrodes: (d) and (e) without field, (f) and (g) under applied field strength of 60 mT. SQUID profiles of Ni–Co LDH@CNT at two temperatures: (h) 300 K, and (i) 5 K. (j) ZFC and FCC curves obtained for Ni–Co LDH@CNT at 0 and 60 mT. (k) Electrochemical performance reversibility comparison plots of Ni–Co LDH@CNT electrode at three different conditions.

In a conventional electrochemical system, slow diffusion of species to the electrode surface is a limiting factor of the reaction rate. When a perpendicular magnetic field is applied to the electrode–electrolyte (KOH) interface as shown in Figure 5a‐(left), the interaction between electric current and the field generates a Kelvin force, inducing MHD convection [11, 18]. This magnetically driven fluid motion enhances transport of electroactive species, reduces diffusion layer (*δ*_d‐b_ ) and forms a new, thinner hydrodynamic boundary layer (*δ*_h_ ), yielding more uniform ion distribution at Ni–Co LDH or Ni–Co LDH@CNT electrodes. Further, under the influence of a perpendicular magnetic field, electrons follow helical trajectories, improving OH^−^ diffusion kinetics and compressing the Nernst layer, increasing local OH^−^ concentration, and lowering charge–transfer resistance. The Lorentz force further induces bulk MHD convection, decreasing electrolyte and interfacial resistances while driving OH^−^ ions deeper into LDH interlayers, thus boosting ion accessibility and capacitance. Therefore, under no applied magnetic field condition, the reaction pathway has a higher activation energy, slowing down redox kinetics. However, when a magnetic field is applied, the energy barrier lowers, indicating enhanced charge transfer [11, 18] as seen from Figure 5a (bottom‐right). The scheme in Figure 5a (top‐right) also illustrates the spin polarization of electrons in Ni–Co LDH‐based electrodes under an external magnetic field. The Ni–Co LDH exhibits an intrinsic ferromagnetic nature from the electronic configuration and magnetic interactions of the transition‐metal ions within the layered framework. Specifically, unpaired d‐electrons in Ni^2+^ and Co^2+^/Co^3+^ ions generate localized magnetic moments that interact via super exchange mediated by hydroxide (OH^−^) bridges. Upon application of an external magnetic field, a parallel spin alignment is obtained, resulting in spontaneous magnetization within the brucite‐like layers. Additionally, the coexistence of mixed valence states (Ni^2+^/Ni^3+^ and Co^2+^/Co^3+^) facilitates electron hopping, further strengthening magnetic coupling and stabilizing the ferromagnetic ordering. These unpaired 3d electrons of Ni and Co respond to the magnetic field, which lifts spin degeneracy via the Zeeman effect, resulting in the splitting of the spin energy levels. The electron's energy associated with magnetic moment can be given as, *gμ*_B_ *H*, where *g* is the *g*‐factor (Lande *g*‐factor), *μ*_B_ and *H* represents the Bohr magneton and applied magnetic field strength respectively. So, here, a higher applied magnetic field strength can result in greater splitting of energy levels, which will alter the electron behavior. The overall energy state and spin alignment of the electrons are promoted by the splitting, thereby reducing spin scattering and enhancing electron mobility [22, 31].

Further, the conductive CNT framework facilitates efficient electron transport, while the applied magnetic field contributes to improved mass transport within the electrolyte. In the hybrid material, the low conductivity of Ni–Co LDH is balanced with the high electrical conductivity of CNTs, enabling rapid electron transfer between active redox sites and the current collector. The CNTs structural scaffold improves the dispersion of LDH nanosheets, increases accessible surface area, and reduces charge transfer resistance, thereby facilitating faster electrochemical kinetics and collectively resulting in superior electrochemical behavior relative to the pristine LDH.

To understand the structural, chemical and physical changes associated with electrochemical behavior due to without/with field conditions, ex‐situ XRD, Raman, FE‐SEM and SQUID analysis were conducted on the Ni–Co LDH@CNT electrodes after cycling under 0 and 60 mT field strengths. The XRD patterns (Figure 5b) show distinct structural differences between the Ni–Co LDH@CNT electrodes cycled under 0 and 60 mT field strength. The characteristic diffraction peaks of Ni–Co LDH are seen in both samples, confirming the existence of the hydrotalcite structure. The XRD pattern also confirms the formation of additional phases due to the coexistence of Co_3_O_4_ (Ref. code: 00‐001‐1152), and CoO (Ref. code: 01‐089‐2803). During electrochemical cycling, partial oxidation and structural reconstruction led to the additional phases. Electrodes cycled under the 60 mT magnetic field condition exhibited strong diffraction peak intensities, confirming enhanced crystallinity due to structural ordering and preferential orientation of the LDH planes. Also, there is a higher angle peak shift by ∼1°–2°, which suggests that an interlayer d‐spacing contraction and a lattice compression, as predicted by Bragg's law. This behavior suggests stronger interlayer interactions and a more compact lattice arising from magnetic‐field‐assisted structural reorganization. Overall, the synergistic effect of electrochemical cycling with a magnetic field can cause the structural modification by promoting in situ reorganization and densifying the layered framework.

The structural evolution of the material was confirmed through Raman spectroscopy (Figure 5c). The electrodes cycled under both 0 and 60 mT magnetic fields exhibit the characteristic D and G bands, corresponding to the disordered carbon structure and graphitic domains of CNTs, respectively. Deconvolution of the Raman spectra in the 1200–1700 cm^−1^ region provided detailed insight into the structural modifications induced by the magnetic field. For the Ni–Co LDH@CNT electrode cycled under 0 mT, an adjusted *R* ^2^ value of 0.9939 revealed that the D and G bands were centered at 1348 and 1603 cm^−1^, respectively. In contrast, the electrode cycled under a 60 mT magnetic field showed D and G bands at 1326 and 1578 cm^−1^, respectively, with an adjusted *R* ^2^ value of 0.9877, confirming reliable peak fitting in both cases. The observed downshift of both Raman bands indicates changes in the local bonding environment, electronic structure, and lattice strain within the CNT network, suggesting that the applied magnetic field influences the carbon framework during electrochemical cycling.

While the pristine CNT exhibited a relatively high *I*_D_/*I*_G_ ratio of 1.53 (Figure S15), the hybrid electrodes showed a notable increase in defect density, as evidenced by the increase in the *I*_D_/*I*_G_ ratio from 0.99 (0 mT) to ∼1.38 (60 mT). This demonstrates that the external magnetic field induces structural disorder during electrochemical cycling. This increase indicates the generation of additional defect sites and local structural disorder within the CNT framework under the applied magnetic field, collectively contributing to enhanced electrochemical activity.

The FE‐SEM images in Figure 5d,e show the morpho‐kinetic evolution of Ni–Co LDH@CNT electrode after electrochemical cycling without magnetic field influence, which display surface features same as the original material. However, the electrode cycled under 60 mT (Figure 5f,g) shows significant morphological changes due to the nucleation, growth, and orientation of the magnetic particles in the electrode matrix. This change is expected because of the interaction between the applied magnetic field and the electronic structure of the material, which causes the alignment of electron spins and affects local magnetic moments. This interaction fundamentally changes bonding dynamics and promotes favorable structural transitions in the material.

To understand the effect of the external magnetic field on the material's magnetic properties, SQUID was used to measure the cycled Ni–Co LDH@CNT electrodes at 0 and 60 mT. The ex‐situ magnetic measurements were performed at 300 K (Figure 5h) and 5 K (Figure 5i) by varying the field in the range from −50 to +50 kOe, revealing soft ferromagnetic behavior for both the samples cycled without field (0 mT) and field (60 mT). The results from SQUID measurements are analyzed and presented in Table 3. The M‐H loops exhibit very low *H*_c,_ and it is further improved for a 60 mT cycled electrode and negligible remanence (*M*_r_ *M*_s_ < 0.17% for 60 mT and 0.10% for 0 mT) indicating nearly reversible magnetization with minimal hysteresis. The quasi‐linear nature of the curves suggests that the magnetic response has a weak ferromagnetic contribution. Notably, the 60 mT cycled sample shows a higher *M*_s_ compared to the electrode cycled under 0 mT, along with a slight increase in *H*_C_ and *M*_r_. At low temperature (5 K), the electrode cycled under 60 mT shows ∼21.4% increase in the value of *H*_c_ in comparison to the 0 mT sample. Similarly, *M*_r_ rises from 2.02% to 2.5%, indicating enhanced magnetic ordering under the applied field.

**TABLE 3:** Magnetic property comparison of Ni–Co LDH@CNT cycled at 0 and 60 mT.

Magnetic properties	Ni–Co LDH@CNT 0 mT		Ni–Co LDH@CNT 60 mT	
	5 K	300 K	5 K	300 K
Coercivity (Oe)	154	17.10	187	23.7
Remanence (emu g^−1^)	0.274	0.0015	0.409	0.0029
Magnetic saturation (emu g^−1^)	13.50	1.452	16.39	1.741

The field cooled condition (FCC) and zero field cooled (ZFC) magnetization curves of the Ni–Co LDH@CNT electrodes cycled at 0 and 60 mT were measured at 500 Oe, showing a large magnetization at low temperature that decreases monotonically with increasing temperature. As shown in Figure 5j(top), the FCC magnetization improved from 0.85 to 1.06 emu g^−1^ for the electrode cycled under a 60 mT field. At 5 K, ZFC magnetization for the 60 mT cycled electrode increased from 0.77 to 0.85 emu g^−1^ as shown in Figure 5j(bottom). The increase in both FCC and ZFC magnetization after cycling under a magnetic field confirms that the field induces more stable and better‐aligned magnetic domains, enhancing the material's magnetic ordering. This improved magnetic structure is often linked to better charge transport and ion diffusion, supporting the observed enhancement in electrochemical performance. The small bifurcation between FCC and ZFC indicates weak magnetic irreversibility associated with weakly interacting magnetic moments arising from sparsely coupled or weakly interacting magnetic moments, common in dilute nanomaterial systems, and FCC magnetization is higher than ZFC, indicating ferromagnetism in the sample. Around 18 K, all curves tend to merge, showing that thermal energy dominates over anisotropy, and the blocking temperature in this case is broader.

To understand the field reversal effect on the electrodes, CV and EIS were performed in three different conditions; under no applied field (Ni–Co LDH@CNT 0 mT), upon application of 60 mT (Ni–Co LDH@CNT 60 mT) and after removal of the applied field (Ni–Co LDH@CNT 0 mT (Field reversal)). The CV profiles (Figure 5k) and EIS (Figure S16a) of Ni–Co LDH@CNT show a partial reversible enhancement in the redox response under the applied field, with partial retention of the improved behavior even after field removal, indicating a field‐responsive but largely nonpermanent process. This partial irreversibility of the material after cycling under 60 mT can be correlated to the FCC and ZFC curves, as we noticed the material exhibits only a small bifurcation, suggesting its weak magnetic irreversibility. This is why the CV curve doesn’t go back to its initial state even after the field is removed. Also, the M–H curve suggests that the material continues to hold some magnetic property while exposed to an external field, as realized from the magnetic properties obtained for the electrode cycled at 60 mT. These investigations also suggest that the magnetic field does not induce a complete structural regeneration but instead promotes a reversible optimization of the electrode interface and morphology. This aligns with the electrochemical observations, where performance recovery can be attributed to improved charge transport and ion diffusion rather than permanent material repair.

### 2.4 Magneto‐Electrochemical Hybrid Asymmetric Supercapacitor

A proof‐of‐concept asymmetric HSC device was assembled with Ni–Co LDH@CNT and activated carbon (AC), a key strategy to enhance energy and power density. For an optimized device configuration, the active masses of Ni–Co LDH@CNT and AC electrodes are evaluated based on the charge balance equation [57],

(4) $$m^{+}/m^{-}=C^{-}\times \text{Δ}V^{-}/(C^{+}\times \text{Δ}{\text{V}}^{+})$$

**FIGURE 6:**
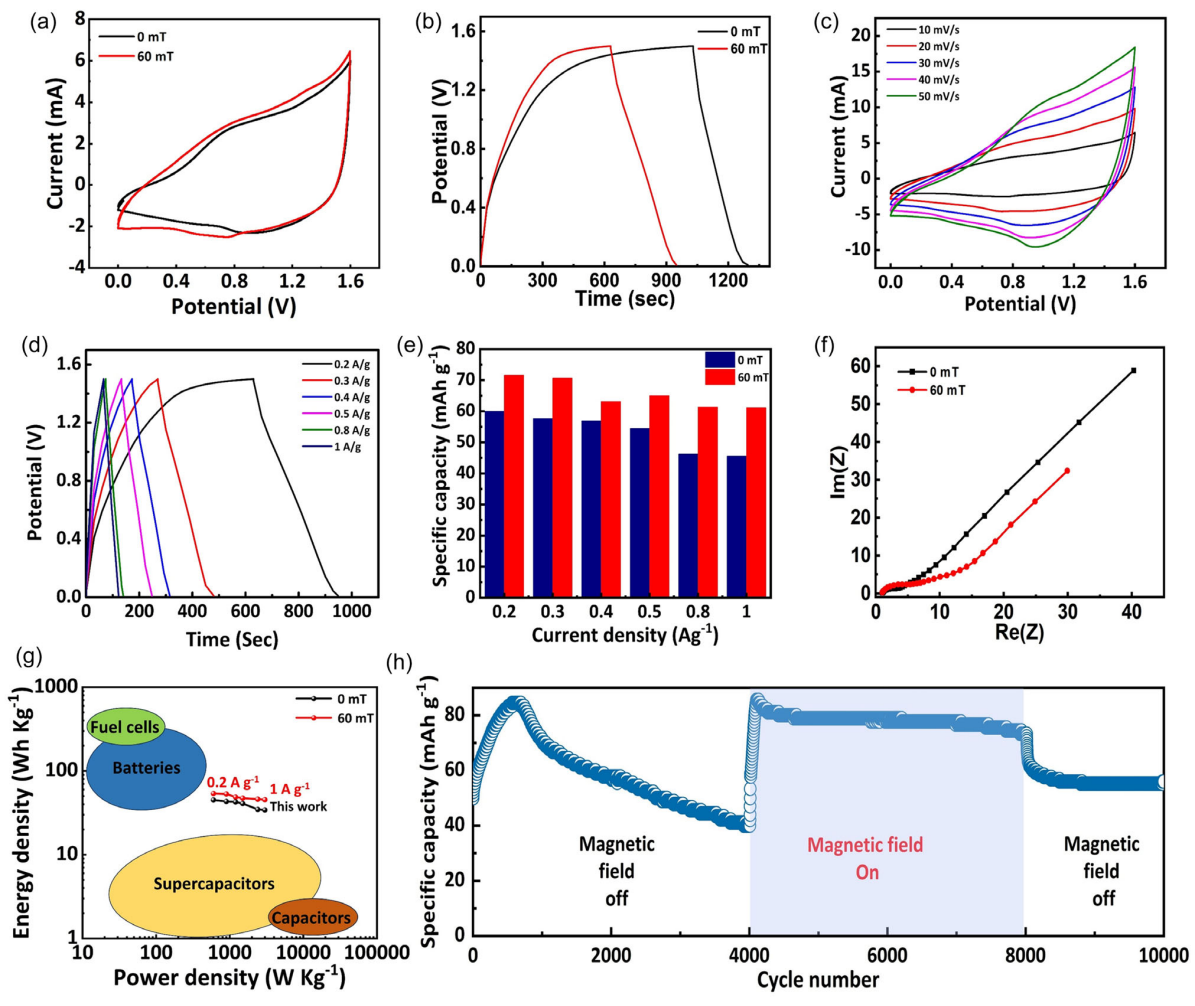
Effect of magnetic field strength on asymmetric hybrid device. (a) CV curves at a scan rate of 10 mV s^−1^. (b) GCD curves at a current density of 0.2 A g^−1^. (c) CV curves of the hybrid device under 60 mT at scan rates of 10–50 mV s^−1^. (d) GCD curves of the hybrid device under 60 mT at current densities in the range of 0.2–1 A g^−1^. (e) Comparison curve of Specific capacity vs. current density. (f) Nyquist plots at 0 mT and 60 mT of magnetic field. (g) Ragone plot. (h) Cycle life stability under magnetic field switching from 0 mT to 60 mT in three intervals.

The asymmetric HSC was evaluated under both the presence (60 mT) and absence (0 mT) of an external magnetic field. At 60 mT (Figure 6a), the CV curves display a larger enclosed area compared to 0 mT, indicating enhanced charge storage performance.

Consistently, the GCD profiles (Figure 6b) recorded at 0.2 A g^−1^ over a field range of 0–60 mT show a significant reduction (∼40%) in charging time under the applied field and increased discharge time at the same time. To further assess the device behavior, scan rate‐dependent CV measurements were performed over a range of 10–50 mV s^−1^ under both conditions, where Figure S17a represents the CV curves at 0 mT and Figure 6c corresponds to those at 60 mT. The peak current increases linearly with scan rates, while the characteristic CV shape is maintained at all scan rates. This indicates a low internal resistance and rapid interfacial charge–transfer kinetics.

Analogously, the GCD curves (Figure S17b and Figure 6d), obtained at 0.2–1 A g^−1^ exhibit a decreasing tendency in discharge time during the applied field condition and display more symmetric charge–discharge profiles with an increasing current density value, indicating that pseudocapacitive behavior is dominant. The specific capacities were obtained from the GCD responses at 0 mT and 60 mT and plotted as bar graphs, as shown in Figure 6e. The charge storage capacity is significantly increased under the applied magnetic field in this device which is clearly observed at all current rates. The specific capacity at 0.2 A g^−1^ is improved from ∼60 mAh g^−1^ (0 mT) to 71.5 mAh g^−1^ (60 mT). Even when cycled at 1 A g^−1^, the cell still retains a higher capacity of 61.1 mAh g^−1^ at 60 mT than that of 45.5 mAh g^−1^ under 0 mT, validating a field‐enhanced capacitance effect. The Nyquist plot (Figure 6f) obtained from 0.1 to 10 kHz reveals that the value of charge–transfer resistance (*R*_ct_) reduces from 4.37 Ω at 0 mT to 1.95 Ω at 60 mT, demonstrating improved ion diffusion due to the MHD effect. Moreover, the corresponding Ragone plot (Figure 6g) under the influence of magnetic field demonstrates that the Ni–Co LDH@CNT//AC device delivers an energy density of 53.66 Wh kg^−1^ with a power density of 600 W kg^−1^, much higher than 45 Wh kg^−1^ under 0 mT at similar power performance. In general, an asymmetric HSC exhibits a higher energy density value than that of the conventional symmetric activated‐carbon systems, but interestingly the magneto‐HSC further exceeds the nonmagnetic devices.

The cycling performance of the asymmetric hybrid device is presented in Figure 6h at a rate of 1 A g^−1^ over 10 000 cycles. For the early 4000 cycles under zero magnetic field, the cell started with an initial capacity of 49 mAh g^−1^ and then the capacity gradually increased until 700 cycles as the electrode underwent an activation process, likely associated with progressive electrolyte infiltration and electrochemical activation of redox‐active sites, followed by gradual capacity fading to approximately 40 mAh g^−1^ till 4000 cycles. By imposition of 60 mT magnetic field from the 4000th cycle, the specific capacity suddenly rises up to 85.5 mAh g^−1^ and maintains remarkably stable during the following cycles. This enhanced specific capacitance is attributed primarily to enhanced ion diffusion, improved charge–transfer kinetics, and electrolyte accessibility induced by magnetically driven convection effects. The field is later removed after 8000 cycles, and for the last 2000 cycles, capacity drastically fades and ultimately remains stable at 55.2 mAh g^−1^. Thus, the reversal of the magnetic field again resulted in capacity decay, confirming the reversible and field‐dependent nature of the enhancement. This obvious reversible shift affirms that the improved electrochemical performance is directly dependent on the magnetic field‐assisted healing (magneto‐healing) behavior of the hybrid electrode. The results emphasize the enormous promise of magnetic‐field responsive device approaches for enhancing the durability, efficiency and long‐term performance. Overall, the results recommend that application of a magnetic field parallel to the electrode surface (Figure S18), as a promising innovation for future batteries and supercapacitors.

## 3 Conclusion

In conclusion, a hollow urchin‐like spiny framework of Ni–Co LDH@CNT has been prepared for a demonstration of the magnetic‐field‐controlled HSC. The local magnetic field gradients produced at the electrode–electrolyte interface results in a high specific capacitance of 1315 mF cm^−2^ (657.8 mC cm^−2^) at 1 mA cm^−2^ under a magnetic field of 60 mT, which is 78.2% increase in specific capacitance (or) capacity than that without the applied field. The Ni–Co LDH@CNT electrode shows high performance, preserving ∼98% of the initial capacitance after 3000 cycles. The improved performance is due to the combined benefits of conductive CNT network and MHD effects, which lead to thinner diffusion layers, faster ion transport and reduced internal resistance. An asymmetric Ni–Co LDH@CNT//AC HSC prototype delivers a maximum energy density of 53.66 Wh kg^−1^ at 600 W kg^−1^ under an external magnetic field of 60 mT, which is higher than the energy density with no field condition (45 Wh kg^−1^). Magnetic field‐responsive stability is evident under 10 000 life cycles testing with only 40 mAh g^−1^ of specific capacity retention for 4000 cycles under zero‐field conditions. Upon switching to 60 mT, a rapid capacity recovery to 85.5 mAh g^−1^ is evident, and the lifecycle stabilized at the highest capacity for the next 4000 cycles, confirming the reversible and field‐dependent nature of the device. This relates to the recovery of the device performance under the influence of a magnetic field and promises the potential of LDH‐based hybrid materials for next‐generation supercapacitors.

## Funding

This study was supported by VIT University (SPL/SG20230142).

## Conflicts of Interest

The authors declare no conflicts of interest.

## Supporting information

Supplementary Material

## Data Availability

The data that support the findings of this study are available from the corresponding author upon reasonable request.
