# Supplementary material for: Magneto‐Responsive Hybrid Layered Double Hydroxides With Improved Electrochemical Performance and Field‐Actuated Healing
Source: Small Sci. 2026 Jul 7;6(7):e70335. doi: 10.1002/smsc.70335 (PMC13344236; doi:10.1002/smsc.70335)
Supplement: Supplementary file 1 — Supplementary Material [file SMSC-6-e70335-s001.pdf]

Supporting Information

Magneto-responsive hybrid LDHs with improved electrochemical performance and field-actuated healing

Jeena Mariya Sebastian[a,b] and Karthik Kiran Sarigamala*[a]

[a]CO_2 Research and Green Technologies Centre, Vellore Institute of Technology, Vellore, Tamil Nadu, 632014, India.
[b]School of Advanced Sciences, Vellore Institute of Technology, Vellore, Tamil Nadu, 632014, India.
*Email: karthikkiran.sarigamala@vit.ac.in

Experimental details

MT-S1: Synthesis of hybrid Ni-Co LDH@CNT

Initially, CNT dispersion was prepared in an alcoholic emulsion containing ethanol and deionised water in 1:1 ratio. The dispersion is sonicated for 30 minutes in an ultrasonic bath. Then $Ni(NO_3)_2.6H_2O$ and $Co(NO_3)_2.6H_2O$ were taken in a feeding ratio of 3:2, and 3.36 g of urea was added and refluxed. Subsequently, the above mixture was transferred to an autoclave and heated for 24 hrs at 120℃. The product from the autoclave was centrifuged and rinsed thoroughly with deionised water and ethanol several times. The obtained product was dried in a vacuum overnight at 60℃. To obtain a pristine Ni-Co LDH sample a similar procedure is repeated without CNTs.

MT-S2 Material characterisation

The as-prepared Ni-Co LDH@CNT powder was characterised using an X-ray diffractometer (Panalytical X Pert3) with Cu Kα radiation (λ =1.5406 Å) over a 2θ range of 5-80° to determine structural properties. Raman spectroscopy (Cora 5001 DUAL) was performed to analyse the materials' vibrational modes**.** The oxidation states of the constituent elements were analysed using X-ray photoelectron spectroscopy (XPS) (Thermo Scientific, K-Alpha-KAN9954133), with spectral fitting performed using Origin software. Magnetic measurements, including magnetisation versus magnetic field (M-H) curves were obtained using a vibrating sample magnetometer (VSM) (Lake Shore, 7404). Microstructural and elemental analyses were conducted using field emission scanning electron microscopy (FE-SEM) and energy-dispersive X-ray spectroscopy (EDX), respectively (Thermo Fisher, FEI QUANTA 250 FEG). Internal nanostructure, lattice fringes, and crystallinity of the material were analysed using High Resolution Transmission Electron Microscopy (HR-TEM) (JEM-2100Plus). The FTIR (IRAffinity-

1) spectrometer was used to identify the functional groups of the samples. Superconducting Quantum Interference Device (SQUID) (SQUID-MPMS3) was used to study the magnetic property of the cycled electrodes from the M-H curve. The sample was first cooled to 5 K in the absence of an external magnetic field and then warmed to 300 K in the presence of an external magnetic field, and the magnetisation was recorded as ZFC. The FCC measurements were done after ZFC measurements, and the magnetisation was recorded while cooling the sample down from 300 K to 5 K with a 500 Oe field.

MT-S3 Electrode fabrication and electrochemical characterisations

The hybrid and pristine electrodes were fabricated with the composition of the active material (Ni-Co LDH@CNT or Ni-Co LDH: 70%), polyvinylidene difluoride (PVDF: 10%), and activated carbon (Super P: 20%). This mixture was dissolved in a suitable amount of N-methyl-2-pyrrolidone (NMP) and mechanically grinded to get a homogeneous slurry. The slurry was coated on to Ni foam (1×1 cm^2) electrodes and transferred to an oven for drying at 80 °C for 12 h in a vacuum oven. The Pt wire and Hg/HgO electrodes were used as counter and reference electrode respectively and aqueous 3 M KOH was used as electrolyte. All the electrochemical investigations were carried out using a potentiostat/galvanostat (VersaSTAT 3).

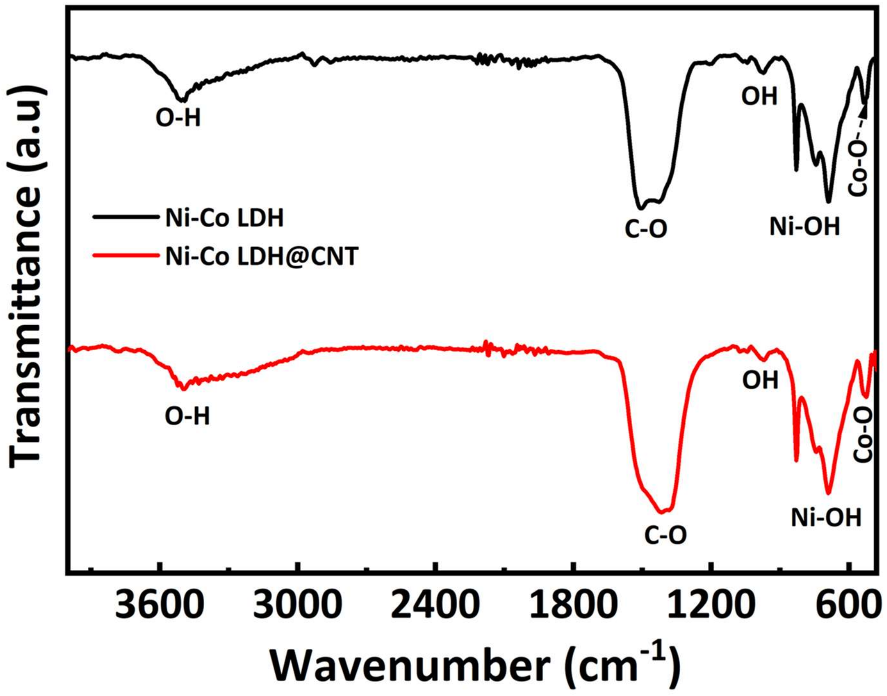

Figure S1. FTIR spectra of Ni-Co LDH and Ni-Co LDH@CNT.

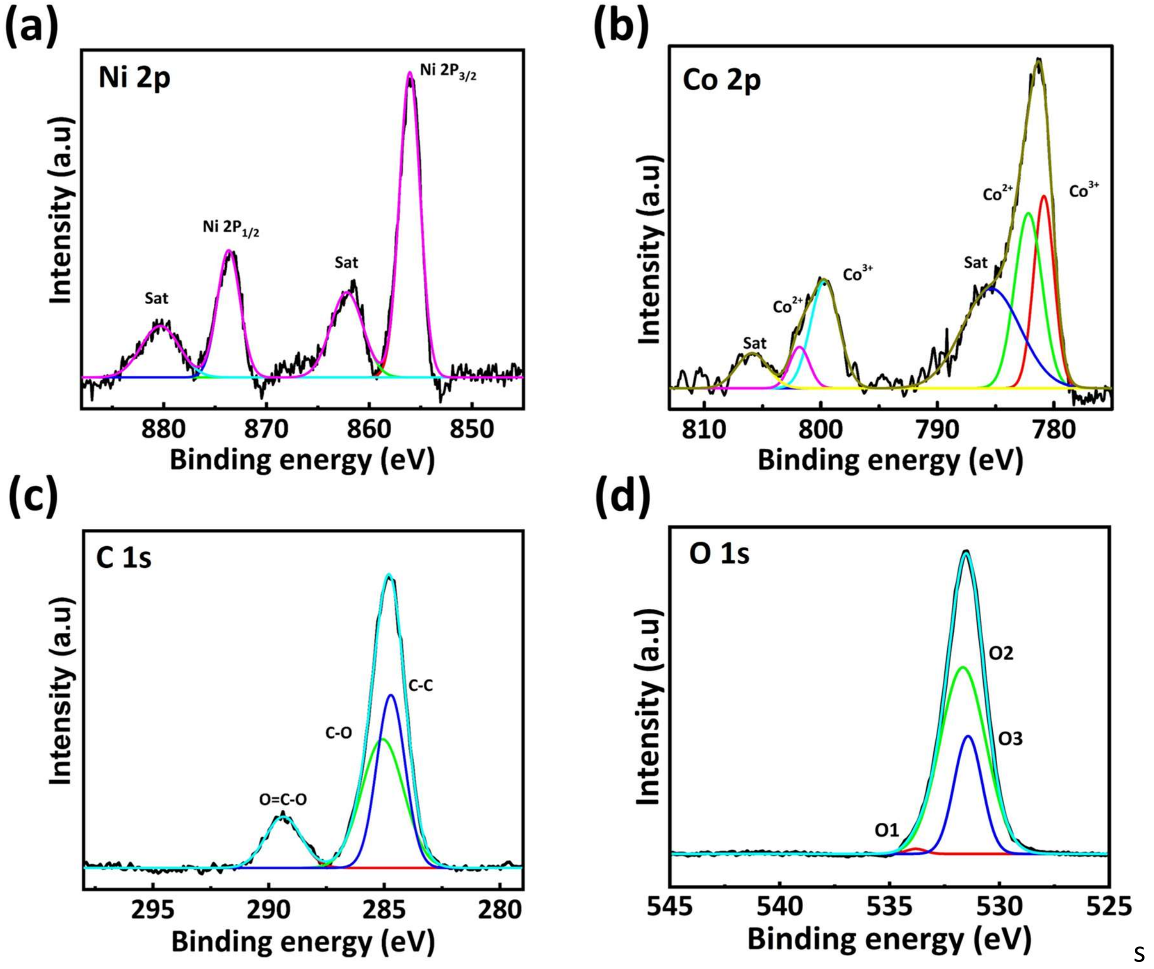

Figure S2. Deconvoluted XPS spectra of pristine Ni-Co LDH: (a) Ni 2p, (b) Co 2p, (c) C 1s, and (d) O 1s.

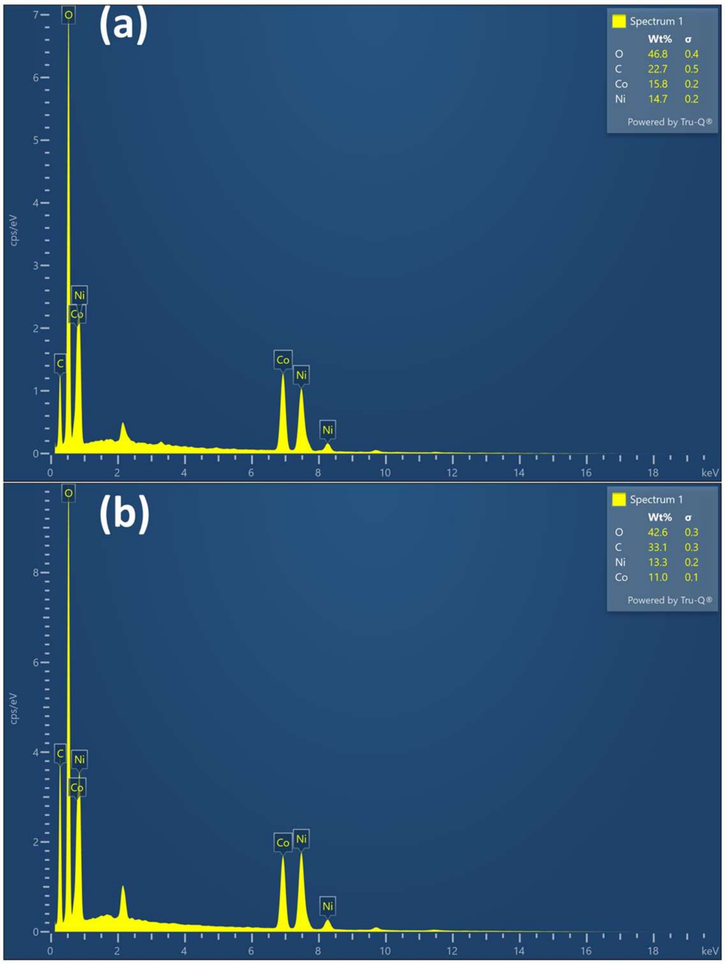

Figure S3. EDS spectrum of (a) Ni-Co LDH, and (b) Ni-Co LDH@CNT.

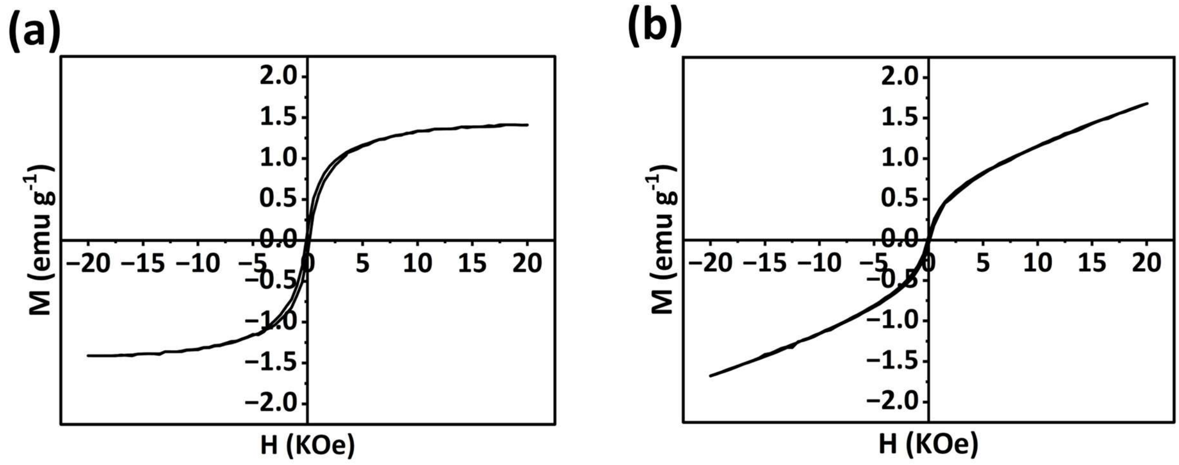

Figure S4. Magnetisation vs. magnetic field intensity (M-H) curve of (a) CNT and, (b) pristine Ni-Co LDH at room temperature.

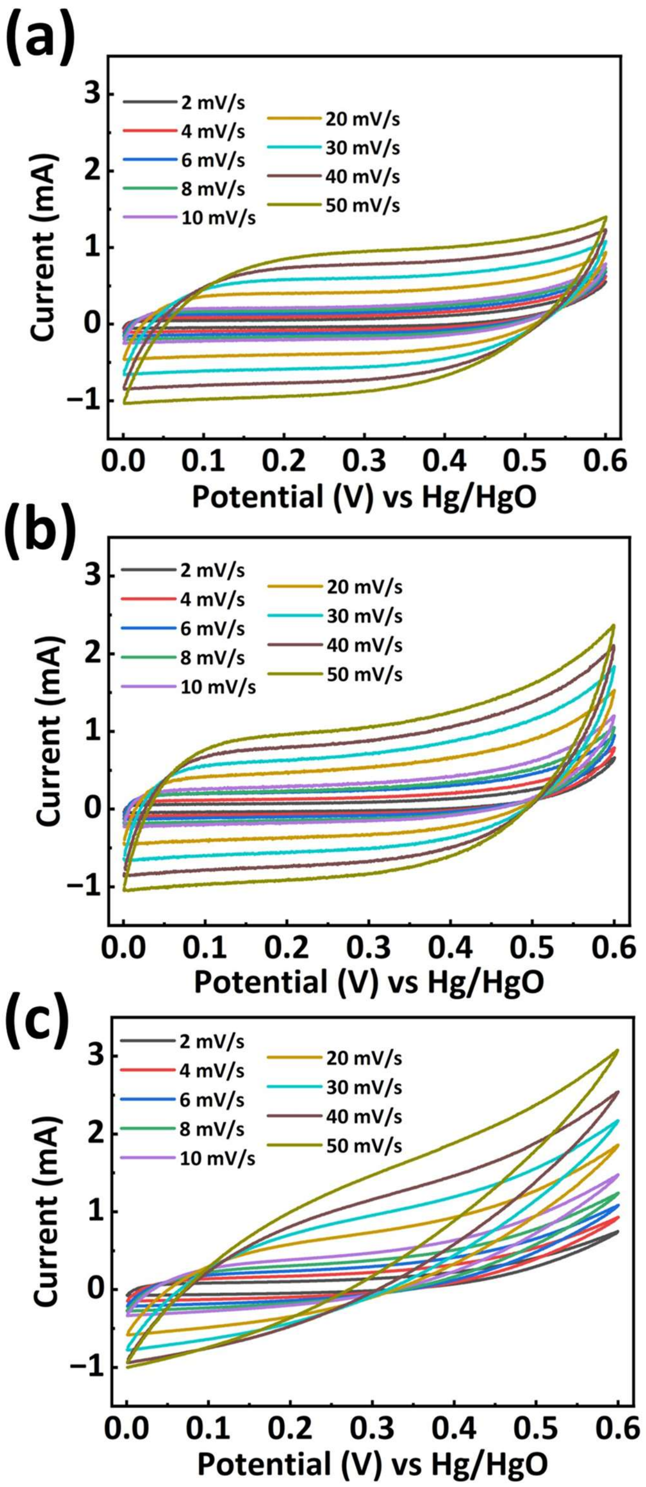

Figure S5. CV curves of CNT at various magnetic field strengths: (a) 0 mT, (b) 30 mT, and (c) 60 mT

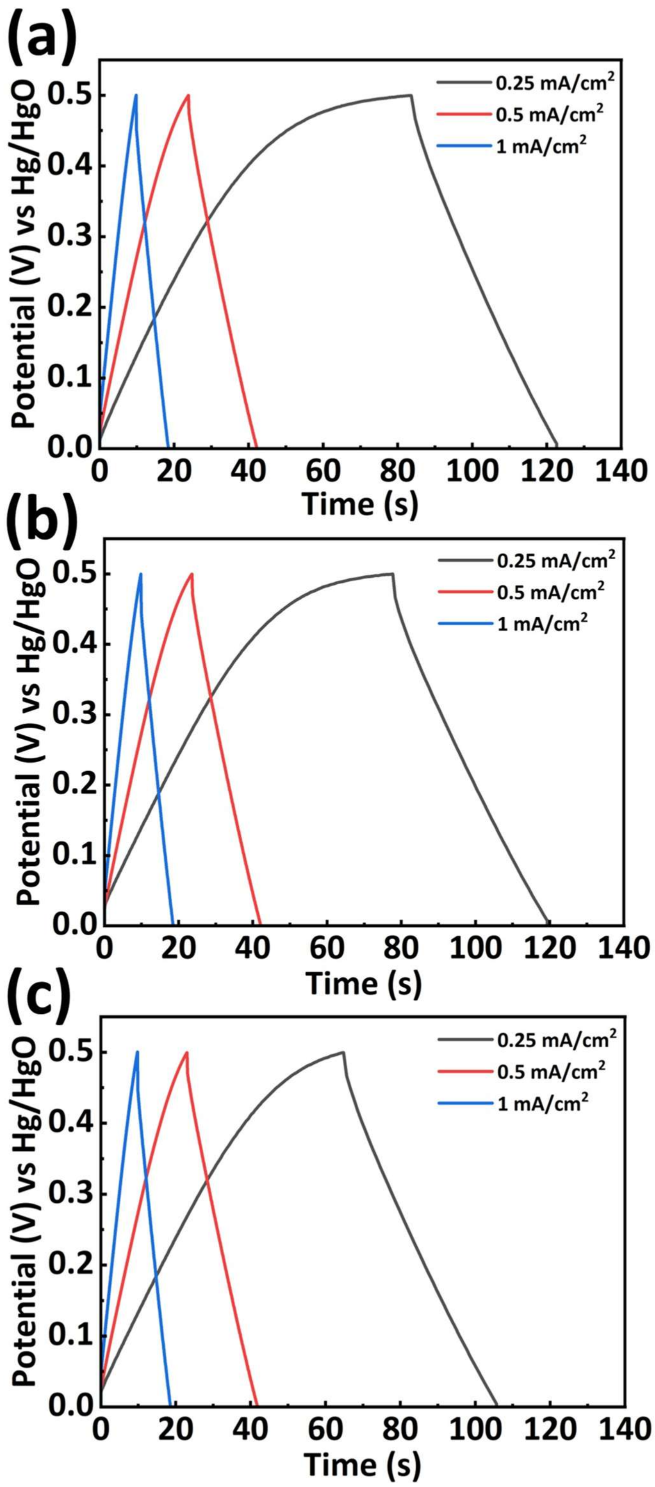

Figure S6. CD curves of CNT at various magnetic field strengths: (a) 0 mT, (b) 30 mT, and (c) 60 mT

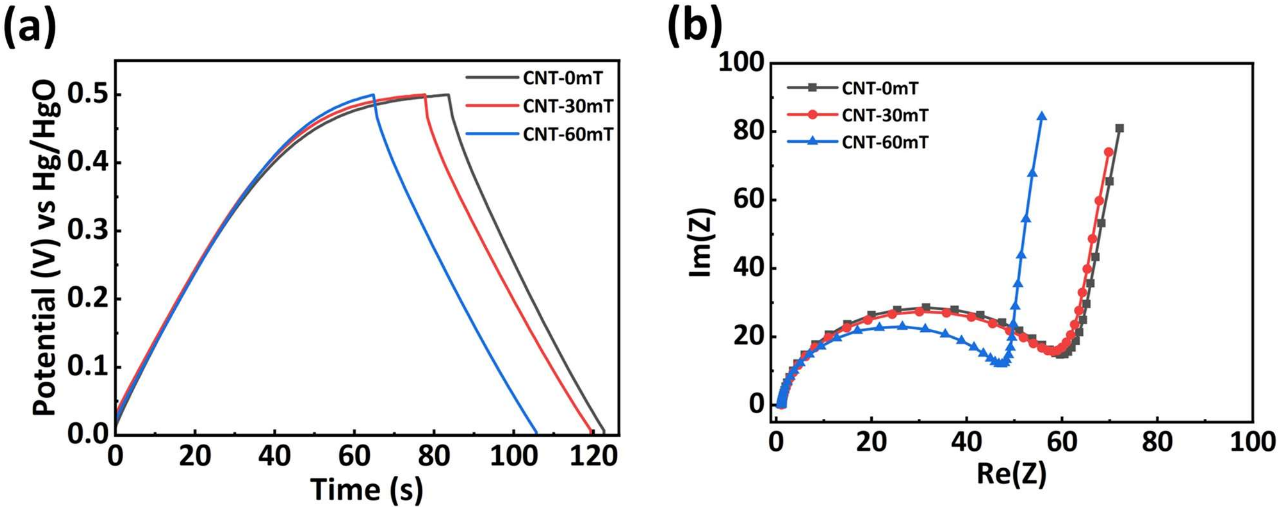

Figure S7. Comparison of electrochemical performance of CNTs at 0 mT, 30 mT, and 60 mT magnetic field strengths: (a) CD curves, and (b) EIS plot.

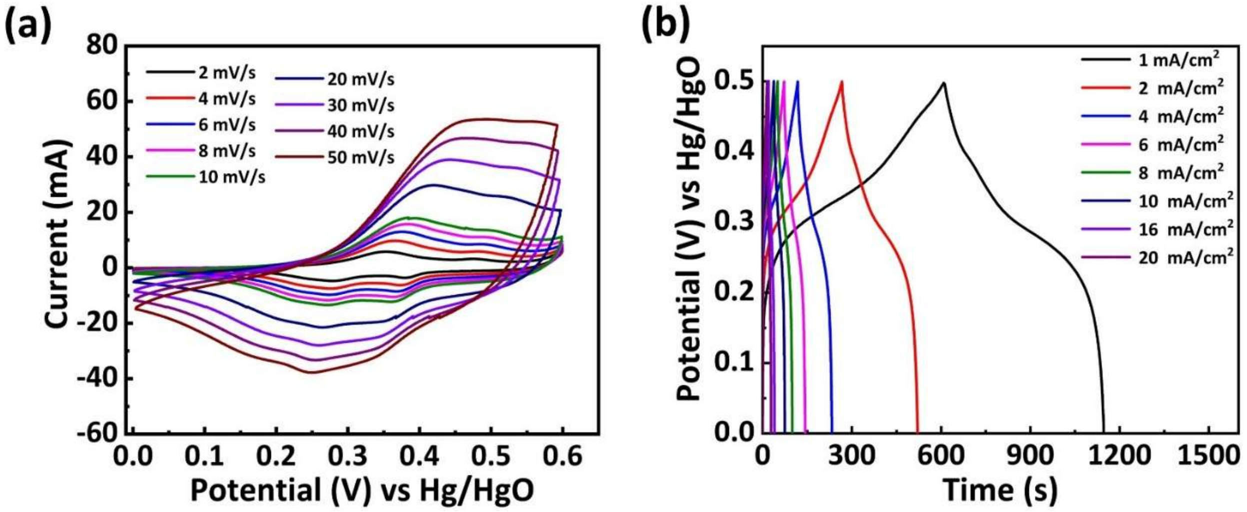

Figure S8. Electrochemical performance of Ni-Co LDH@CNT at 30 mT field strength: (a) CV curves, and (b) CD curves

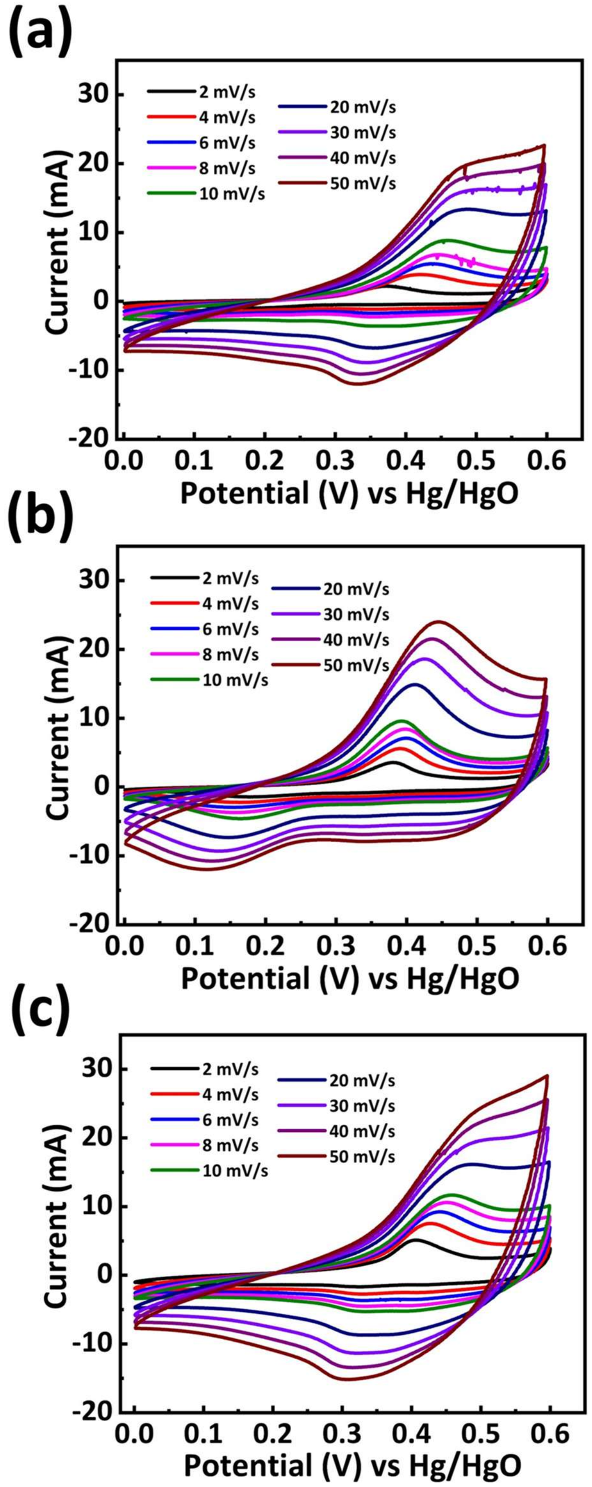

Figure S9. CV curves of Ni-Co LDH at three magnetic field strengths: (a) 0 mT (b) 30 mT, and (c) 60 mT.

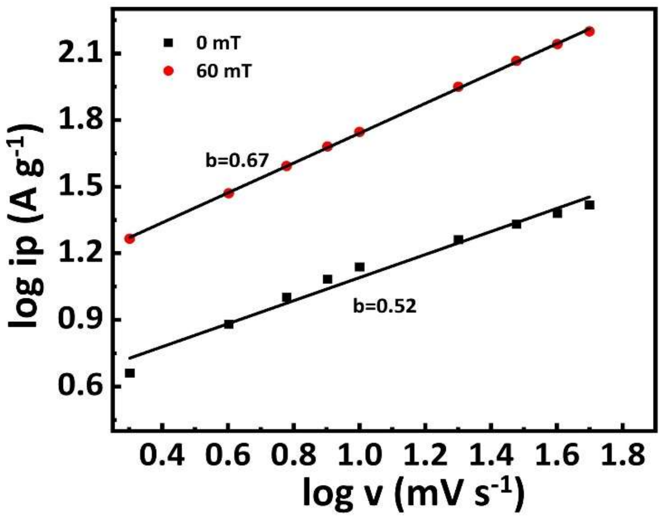

Figure S10. Log v *vs*. log i_p plot of Ni-Co LDH obtained at magnetic field strengths of 0 mT and 60 mT.

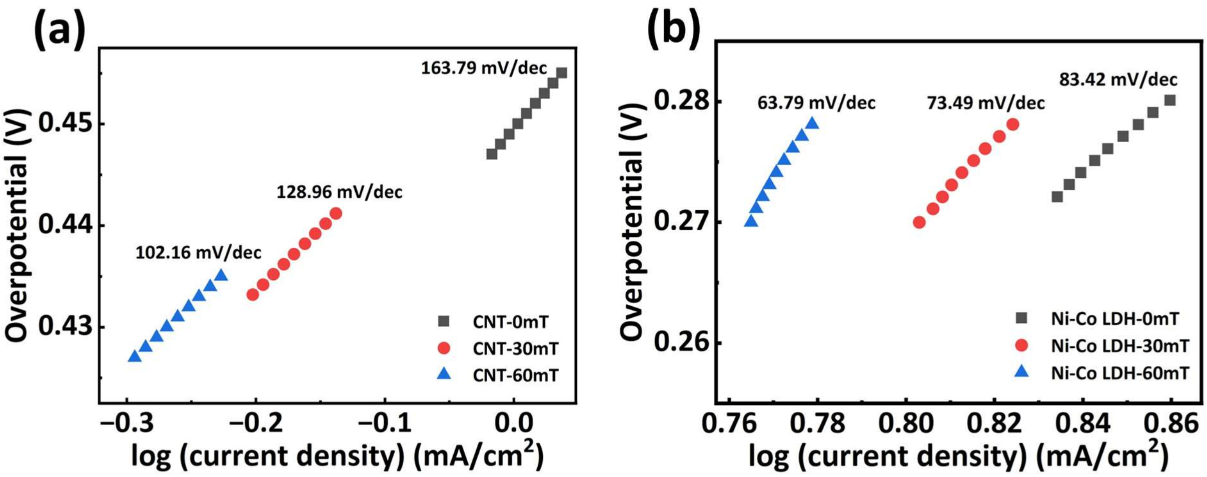

Figure S11. Tafel slopes: (a) CNT, and (b) Ni-Co LDH

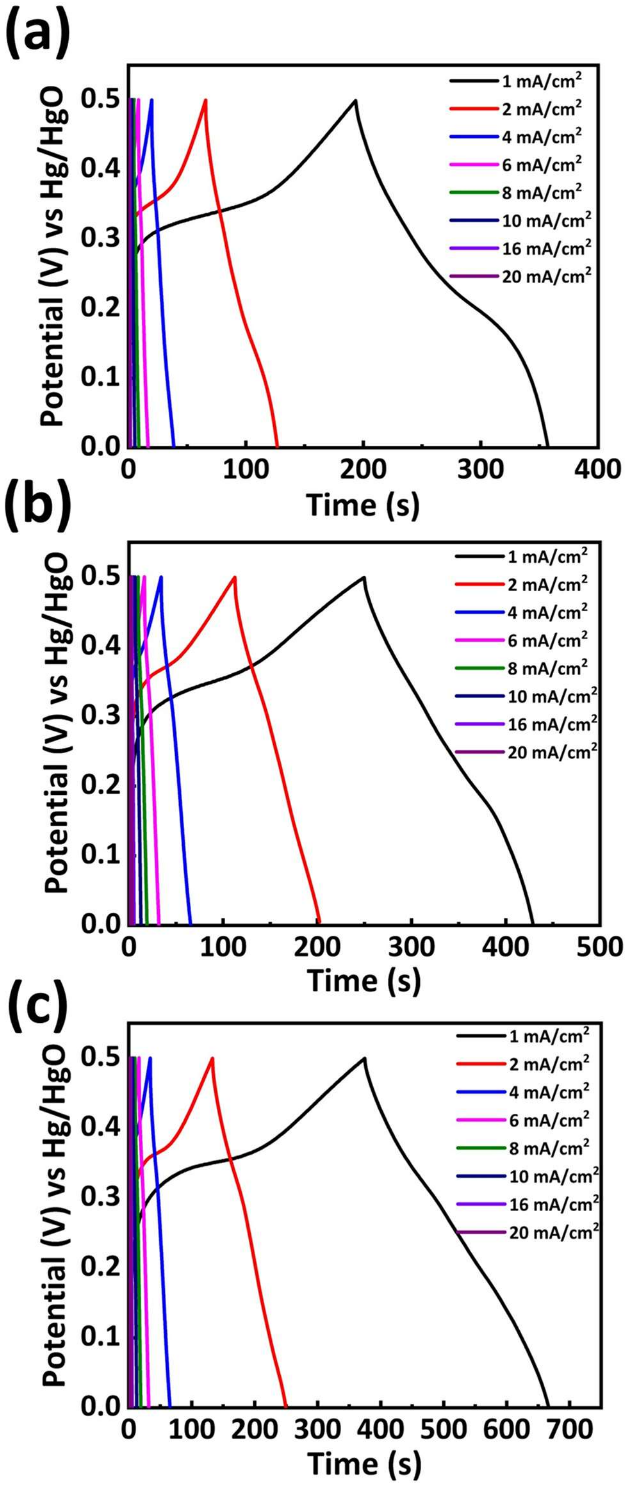

Figure S12. CD curves of Ni-Co LDH obtained at three magnetic field strengths: (a) 0 mT, (b) 30 mT, and (c) 60 mT respectively.

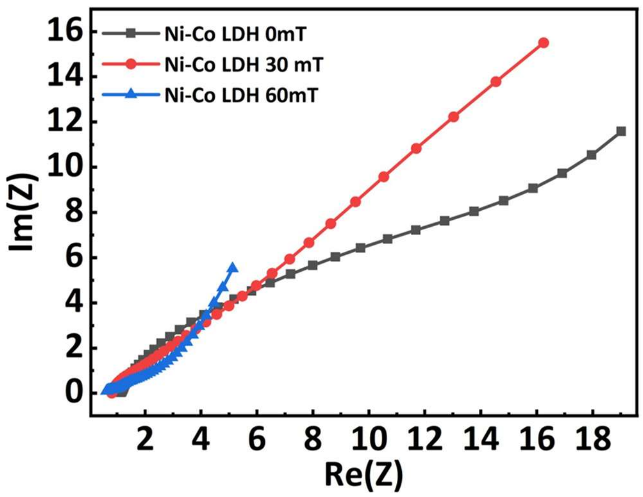

Figure S13. EIS profiles of Ni-Co LDH obtained at three magnetic field strengths of 0 mT, 30 mT, and 60 mT respectively

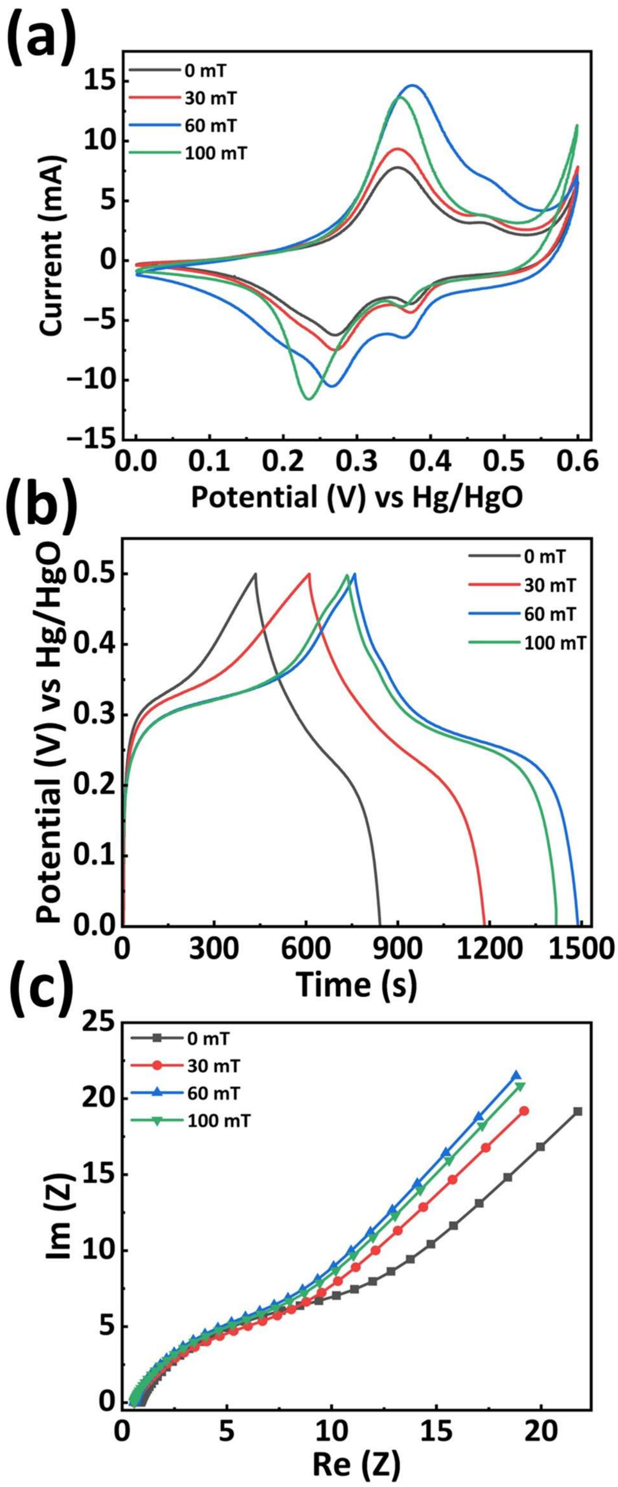

Figure S14. Electrochemical performance comparison of Ni-Co LDH@CNT electrode obtained at four different magnetic field strengths of 0 mT, 30 mT, 60 mT, and 100 mT respectively: (a) CV curves, (b) CD curves, and (c) EIS plots.

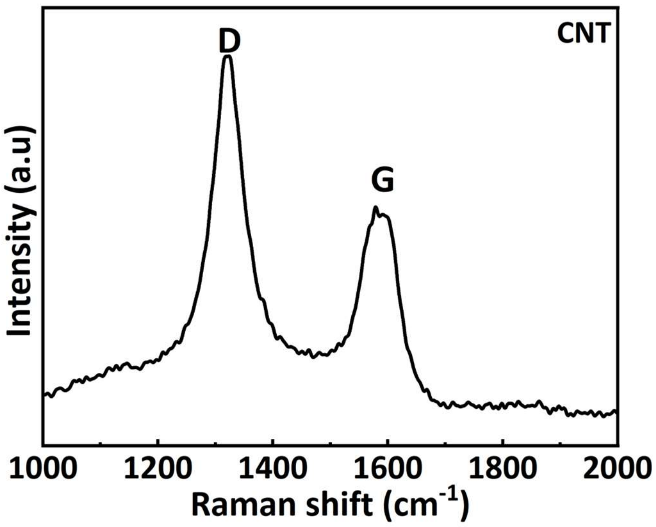

Figure S15. Raman spectra of CNT.

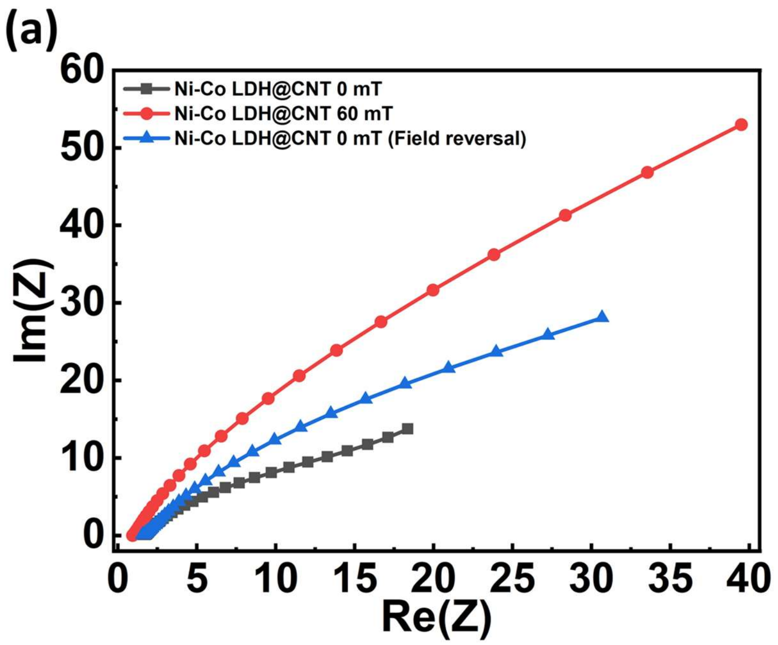

Figure S16. EIS plot showing electrochemical reversibility test for Ni-Co LDH@CNT electrode at 3 different conditions: no field (Ni-Co LDH@CNT 0mT), upon application of 60 mT (Ni-Co LDH@CNT 60mT) and after removal of the applied field (Ni-Co LDH@CNT 0mT (Field removal))

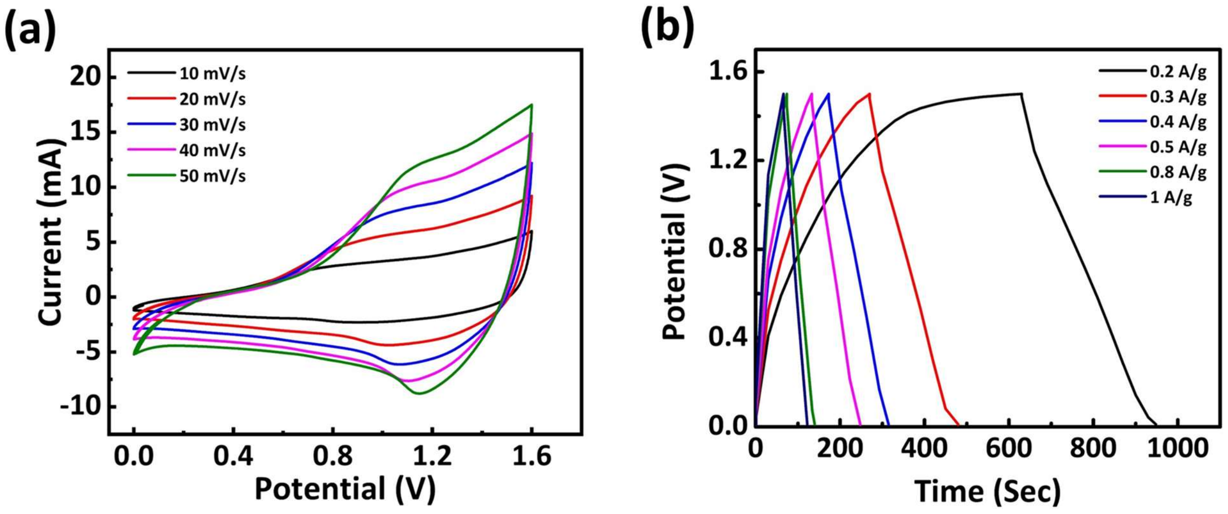

Figure S17. Electrochemical performance of Ni-Co LDH@CNT//AC hybrid device obtained under 0 mT field strength condition: (a) CV curves at various scan rates, and (b) CD curves at various current densities.

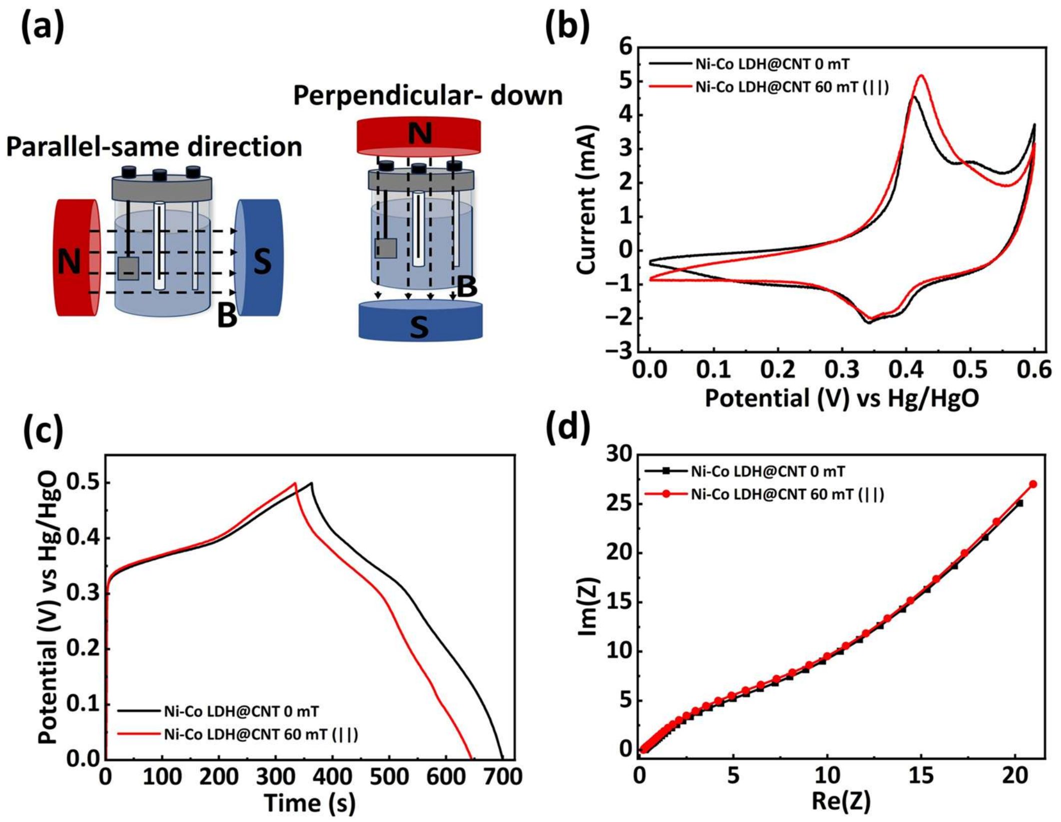

Figure S18. (a) Schematic illustrating the field alignment with electrode: Parallel same direction and Perpendicular-down field arrangement. Electrochemical performance of the (Ni-Co LDH@CNT in perpendicular down orientation: (b) CV profiles, (c) CD curves and (d) Nyquist plot.

Table S1: Electrochemical performance comparison of different electrode materials under the influence of an external magnetic field.

Material	Synthesis method	Electrolyte	Operating Window (V)	Field strength (mT)	Capacitance		Current Density	Percentage enhancement (%)	Stability	Ref
					Without field	With field				
Fe_2O_3	Forced hydrolysis and reflux	1 M KOH	0.95	5	86.05 F g^{-1}	133.3 F g^{-1}	1 A g^{-1}	54.91	91% over 1000 cycles	1
$FeCo_2O_4$	Electrospinning	2 M KOH	0.65	3	80 F g^{-1}	111 F g^{-1}	0.25 A g^{-1}	38..75	93±2% over 2000 cycles	2
Co_3O_4	Electrospinning	2 M KOH	0.45	3	188 F g^{-1}	200 F g^{-1}	0.25 A g^{-1}	6.38	-	3
MnO_2/ECNFs	Electrospinning	6 M KOH	0.8	1.34	119.2 F g^{-1}	141.7 F g^{-1}	5 mV s^{-1}	18.87	90.6 % over 1000 cycle	4
NCOH	Hydrothermal	3 M KOH	0.55	100	709.3 C g^{-1}	879.1 C g^{-1}	1 A g^{-1}	23.96	-	5
Ni-Co LDH	Hydrothermal	3 M KOH	0.6	60	303 mF cm^{-2}	535 mF cm^{-2}	1 mA cm^{-2}	76.57	3000 cycles	**This work**
**Ni-Co LDH@CNT**	Hydrothermal	3 M KOH	0.6	60	738 mF cm^{-2}	1315 mF cm^{-2}	1 mA cm^{-2}	78.19	98 % over 3000 cycles	

References

[1] A. Chowdhury, A. Dhar, S. Biswas, V. Sharma, P. S. Burada, A. Chandra, "Theoretical Model for Magnetic Supercapacitors-From the Electrode Material to Electrolyte Ion Dependence," *Journal of Physical Chemistry C* 124, (2020): 26613-26624.

[2] M. Singh, A. Sahoo, K. L. Yadav, Y. Sharma, "Toward the Origin of Magnetic Field-Dependent Storage Properties: A Case Study on the Supercapacitive Performance of FeCo2O4 Nanofibers," *ACS Applied Materials & Interfaces* 12, (2020): 49530-49540.

[3] M. Singh, A. Sahoo, K. L. Yadav, Y. Sharma, "Role of magnetism present in the cobaltites (ACo_2O_4 A = Co, Mn, and Fe) on the charge storage mechanism in aqueous supercapacitor," *Applied Surface Science* 568, (2021): 150966.

[4] Z. Zeng, Y. Liu, W. Zhang, H. Chevva, J. Wei, "Improved supercapacitor performance of MnO_2-electrospun carbon nanofibers electrodes by mT magnetic field," *Journal of Power Sources* 358, (2017): 22-28.

[5] Z. Zhang, C. Li, P. Ding, L. Guan, Z. Li, S. Zhang, D. Xing, J. Tao, "Magnetic field-enhanced crystalline-amorphous hybrid nickel-cobalt hydroxide nanotubes for high-energy and 20,000-cycle stability in supercapacitors: mechanistic insights and performance enhancement," *Electrochimica Acta* 528, (2025): 146287.
